# Diazinon induces testicular dysfunction and testicular cell damage through increased reactive oxygen species production in mouse

**DOI:** 10.1038/s41420-025-02399-8

**Published:** 2025-03-21

**Authors:** Ran Lee, Won-Young Lee, Dong-Wook Kim, Hyun-Jung Park

**Affiliations:** 1 Department of Livestock, Korea National University of Agriculture and Fisheries, Jeonbuk, Korea; 2 Department of Animal Biotechnology, College of Life Science, Sangji University, Wonju-si, Korea

**Keywords:** Endocrine reproductive disorders, Apoptosis

## Abstract

Diazinon (DZN) is an organophosphorus compound used as a pesticide and is an environmentally hazardous substance to which the human body is commonly exposed. In this study, we evaluated the toxicity of DZN to the male reproductive in mice. For in vivo experiments, mice were intraperitoneally injected with 30 mg/kg DZN for 35 days. Microscopic analysis revealed that the diameter of the spermatogonia in the testes decreased, and the number of differentiating germ cells decreased. Sperm motility in mice injected with DZN was reduced, and slow motility was observed. The rate of neck deformation in the sperm increased in DZN-treated mice. The number of germ and Sertoli cells decreased, and the levels of serum testosterone and steroidogenesis markers also decreased in DZN-treated mice. In addition, DZN-induced oxidative stress in the testes. For in vitro experiments, DZN was toxic to GC-1 spermatogonia and TM4 and TM3 cells derived from mouse testes. DZN generated reactive oxygen species (ROS) and induced mitochondrial dysfunction, suggesting a molecular mechanism underlying ROS-induced cell death. DZN upregulated BAD, cleaved-caspase 3, and phospho-p53 at the cellular level. We also found that this toxicity could be mitigated by N-acetyl-l-cysteine, an ROS inhibitor.

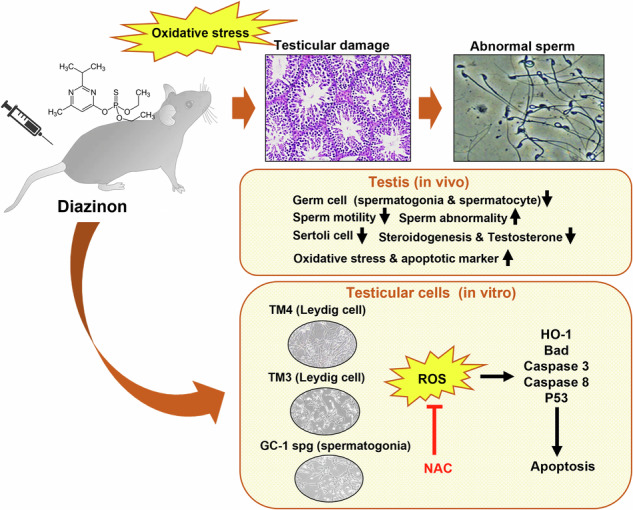

## Introduction

Recently, an increasing trend of infertility due to various environmental issues has been observed worldwide. In males, one crucial reason for infertility is decreased sperm quality, including sperm motility, progressive motility, and count [1, 2]. In recent years, pesticide use has increased owing to agricultural intensification and human convenience; however, these pesticides impact environmental and human health, including decreasing reproductive function [3]. Nevertheless, the excessive use of pesticides is due to their low cost relative to the economic benefits, such as increased agricultural productivity.

Organophosphorus (OP) pesticides, such as diazinon, parathion, and malathion, are widely used in agricultural and residential fields. Diazinon (DZN) is an organophosphate insecticide used in agriculture to control the pests of crops and vegetables and is also used in lawns and gardens [4]. The toxicity of DZN has been investigated in various organs, including the brain, liver, pancreas, and kidneys [5–7]. DZN exposure can result in poisoning, mutagenesis, carcinogenesis, and other adverse effects. In the nervous system of neonates, DZN inhibits acetylcholinesterase, thereby affecting nerve cell development [8]. DZN also induces oxidative stress in rat hepatocytes and damages renal function by decreasing renal glutathione and producing antioxidant enzymes [9, 10].

Several studies have reported the effects of DZN on the reproductive system. Adamkovicova et al. investigated the effects of exposure to Cd and DZN on rat testes and epididymis and found that both the testis and epididymis weights increased after exposure to 40 mg/L DZN for 90 days. In addition, necrosis and degeneration of epithelial cells, remnants of the basement membrane, irregular shape, disarranged epithelial layers, and detached germ cells in the seminiferous tubules of testis were observed in DZN-exposed rats through histopathological analysis [11]. Another study also reported that the testes of DZN-treated rats showed an increase in lipid peroxidation with oxidative stress, serum lactate dehydrogenase, and decreased glutathione and serum testosterone levels [12]. In particular, DZN exposure may lead to an imbalance in reproductive hormones, which are important for the growth, maturation, and development of puberty in humans and animals. DZN administered at 4.1 mg/kg to mice for 4 weeks increased testosterone levels, but it reduced testosterone levels when administered at 8.2 mg/kg [13].

The toxic effects of DZN have also been observed in the female reproductive system. DZN increased the rate of infertility in females and prevented normal ovarian development, including follicle formation, and diseases such as polycystic ovary syndrome (PCOS) [14]. Wang et al. also investigated the reproductive toxicological effects on cultured porcine ovarian granulosa cells. The results indicated that DZN exposure led to excessive reactive oxygen species (ROS) production and DNA damage, ultimately inducing apoptosis and autophagy by inhibiting the PI3K-Akt pathway [15]. During oogenesis, DZN exposure leads to the production of poor-quality oocytes, a major cause of infertility, low pregnancy rates, and high abortion rates [16]. In particular, DZN exposure impairs nuclear and cytoplasmic maturation during oocyte meiosis. Disruption of mitochondrial repositioning and function elevates ROS levels and triggers early apoptosis. DZN-exposed oocytes exhibited reduced Juno expression, resulting in decreased sperm binding compared to normal oocytes. Loss of gap junctions and failure to activate ERK1/2 also contribute to the compromised cytoplasmic maturation; however, research on meiosis regulation by DZN in the male reproductive system is limited [17].

Many researchers have reported mechanisms of DZN toxicity in various cell types. DZN induced oxidative stress and ROS generation in erythrocytes [18], neuronal cells [19], and hepatic cells [20] in mice and in a zebrafish fibroblast cell line [21]. Exposure to DZN or diazoxon did not significantly alter the proliferative capacity of tilapia lymphocytes despite an increase in acetylcholine (ACh) concentration. ACh is expressed not only in neurons but also in other types of tissues. It has also been shown that immune system cells and DZN-induced immunotoxicity produce ACh through the non-neuroma lymphocytic cholinergic system [22]. Regarding the molecular signaling pathway that leads to cellular damage after exposure to OP compounds such as DZN, OP compound exposure activates the ERK, JNK, and p38-MAPK pathways, initiating oxidative stress and apoptosis across diverse tissues [23]. Although studies have been conducted on the effects of DZN and sperm parameters in testis in vivo, the present study is the first to investigate the mechanism of DZN on testes and each testicular cell type.

## Results

### Effects of DZN on the body and testes weights, sperm motility, and sperm dysfunction of mice

**Fig. 1:**
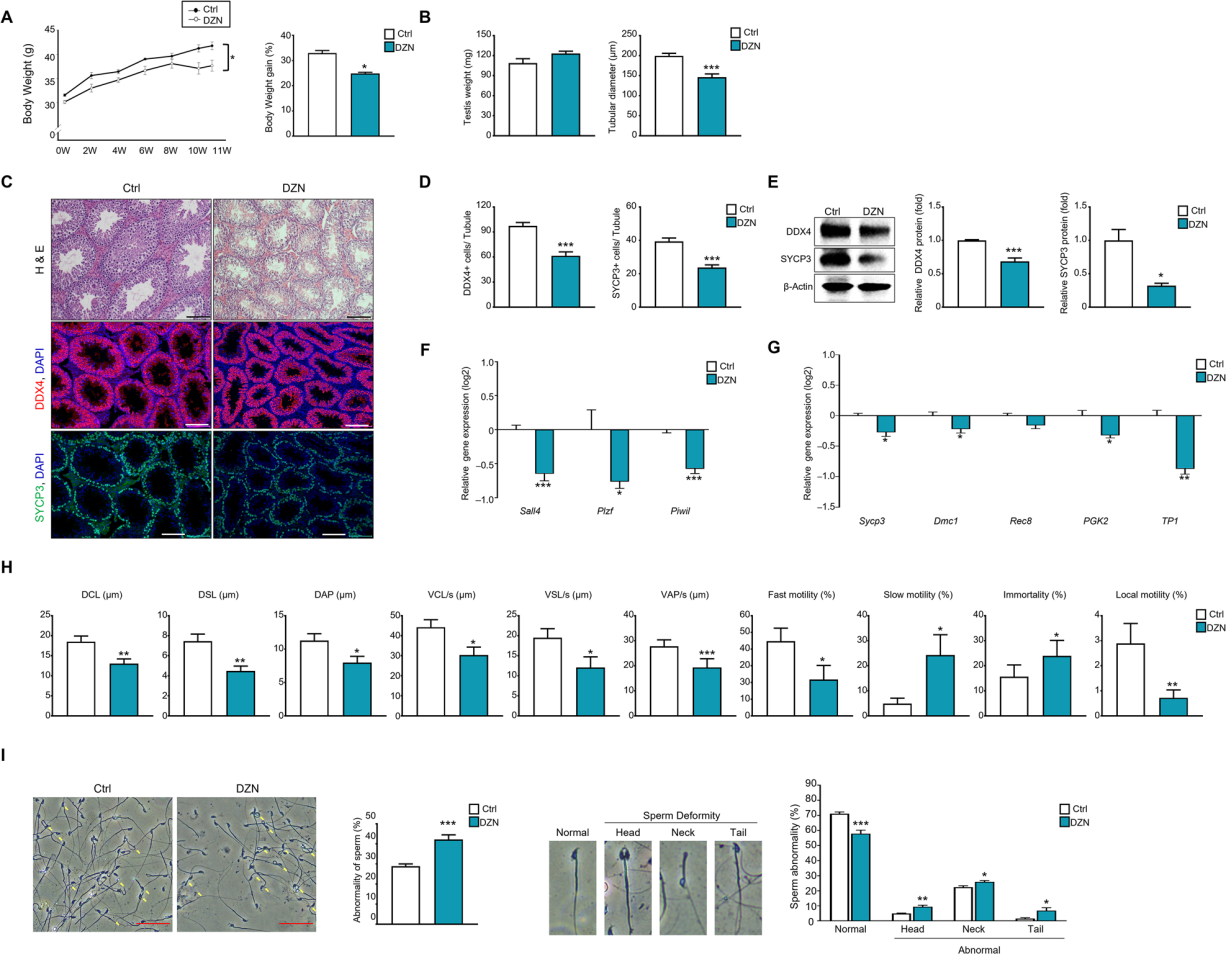
Comparison of body weight changes, intratesticular cell composition, sperm malformations, and motility between DZN-treated and control mice. **A** Body weight changes were measured weekly until the end of the experiment (0–11 weeks). Data are presented as the mean ± standard deviation (SD). **B** Comparison of testicular weight and seminiferous tubule diameter in DZN-injected and control mice. The data is shown as mean ± SD. **C** H&E staining of testes from DZN-injected and control mice, comparison of positive cells of DDX4 and SYCP3 in mouse testes of each experimental group. Scale bar = 100 μm. (**D**) Number of DDX4+ and SYCP3+ cells in testicular tubules. At least 40 tubules were scored for each testis (five to six biological replicates). Values are presented as the mean ± SD. (**E**) Protein expression levels of SYCP3 and DDX4 in testes of DZN-treated and control mice. Graph represents relative protein levels (mean ± SD, n = 5). (**F**) Expression levels of *Sall4, Plzf,* and *piwii* in testes from each group. Values are presented as the mean ± SD (log2 scale). (**G**) Expression levels of *Sycp3*, *Dmc1*, *Rec8*, *Pgk2*, and *Tp1*. Values on the graph are presented as the mean ± SD (log2 scale). (**H**) Comparison of sperm motility and parameter including distance average path (DAP, μm), distance curved line (DCL, μm), distance straight line (DSL, μm), velocity average path (VAP, μm/s), velocity curved line (VCL, μm/s), velocity straight line (VSL, μm/s) and percentage of fast motility, slow motility, local mortality and immortality through CASA analysis. (**I**) Microscopic analysis of sperm from each experiment group. The graph shows the proportion of abnormal structures in the head, tail, and neck of sperm from DZN-treated and control mice. Abnormality of sperm rations was calculated from seven independent experiments and measured 100 spermatozoa at least in each independent experiment. Asterisks indicate significant differences between the treatment and control groups at *p < 0.05, **p < 0.01, and ***p < 0.001. Scale bar = 50 μm. All statistical analyzes for the graphs were conducted using a t-test.

To determine the effect of DZN on the male reproductive system, the body weight, testicular weight, and diameter of seminiferous tubules in mice were measured. Before the experiment, the average weight of mice in the control group was 31.6 g, whereas that of the DZN-treated group was 30.2 g (Fig. 1A). At the end of the experimental period, the DZN-treated group had an average weight of 37.67 g, which is significantly less than that of the control group (41.78 g). Notably, significant weight loss in the DZN-treated group was observed between the 10th and 11th weeks. Six weeks post-injection, the weight of the DZN-treated group was 36.7 g, with no significant weight change up to 11 weeks. The control group gained a significant amount of weight (32.3%) compared with that of the DZN-treated group (24.8%) (Fig. 1A). Regarding testicular weight, there were no significant changes among the experimental groups. However, the testicular diameter in the DZN-treated group (146 ± 1 µm) was significantly reduced compared to the control group (199.3 ± 6 µm) (Fig. 1B).

The morphological changes in seminiferous tubules were assessed using immunostaining for DDX4 and SYCP3 (Fig. 1C). Similar to the testis tubular diameter measurement results in Fig. 1B, histological analysis showed that the tubules of the DZN-treated groups were smaller than those of the control groups. The number of DDX4+ and SYCP3+ cells was counted in each group. DDX4 is a broad germ cell marker with differentiated and undifferentiated germ cells, and SYCP is a meiotic marker in germ cells. The results showed that the control groups had an average of 97.4 DDX4+ germ cells per seminiferous tubule, whereas the DZN-treated group had 61.6, indicating a significant reduction. Additionally, the number of SYCP3+ cells was also reduced in DZN-treated group (23.7) compared to control (39.3) (Fig. 1D). Similarly, immunoblot results revealed significant reductions in DDX4 (30%) and SYCP3 (68%) protein levels in the DZN-treated group compared to the control group (Fig. 1E). Gene expression levels of germ cell markers were detected in each group. mRNA levels of undifferentiation germ cell markers such as *Sall4, Plzf*, and *Piwii* were significantly decreased in the DZN-treated group compared to the control group (Fig. 1F). Differentiation markers, such as *Sycp3, Dmc1*, *Rec8*, and *Transition proteins 1* (*TP1)* also exhibited a significant decreasing trend (Fig. 1G).

Sperm motility parameters, such as distance curved line (DCL, μm), distance straight line (DSL, μm), distance average path (DAP, μm), distance curved line (DCL, μm), velocity curved line (VCL, μm/s), velocity straight line (VSL, μm/s), velocity average path (VAP, μm/s), and percentage of fast motility, slow motility, and immortality were measured for each group. DCL, DSL, DAP, VCL, VSL, VAP, and percentage of fast motility were significantly lower in spermatozoa from the DZN-treated group than that from the control group, whereas the percentage of slow motility and immortality were higher in spermatozoa from the DZN-treated groups (Fig. 1H). Additionally, the DZN-treated group showed an increased incidence of sperm deformities in the head, tail, and neck regions. Specifically, 42.16% of the DZN-treated sperm were malformed, a significant increase compared to the control group (28.9%) (Fig. 1I).

### Effect of DZN Sertoli and steroidogenesis in the testis

**Fig. 2:**
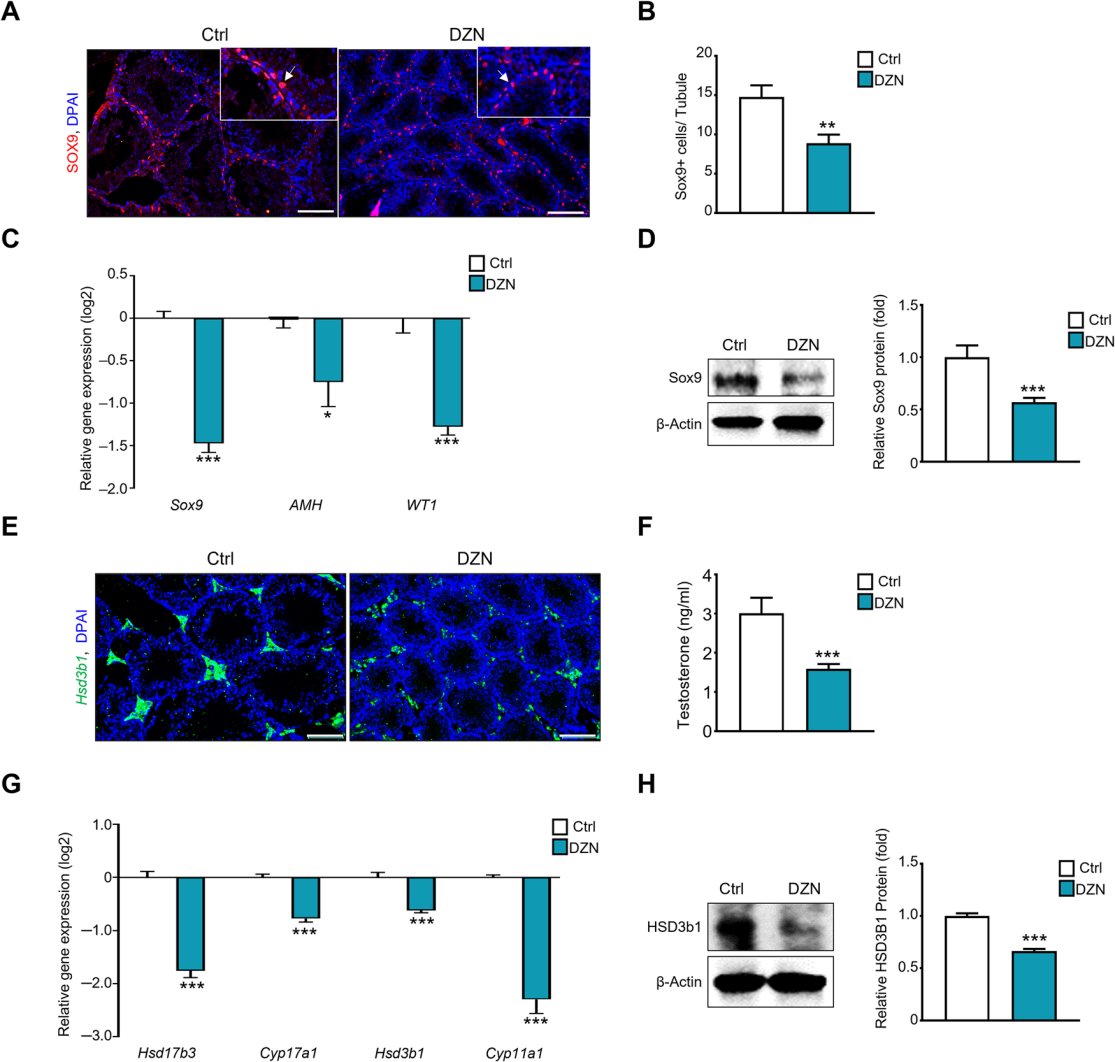
DZN exposure affects Sertoli cell and steroidogenesis in mice testis. **A** Immunostaining of SOX9, a Sertoli cell marker, in testes of DZN-treated and control mice. Scale bar = 100 µm. **B** The number of SOX9+ cells in testicular tubules. At least 40 tubules were scored for each testis (five to six biological replicates). Values on graph are presented as the mean ± SD. **C** Gene expression levels of *Sox9*, *Amh,* and *Wt1* in testis from each group. Values on graph are presented as the mean ± SD (log2 scale). **D** Protein expression levels of SOX9 in testes of DZN-treated and control mice. The graph shows relative protein expression levels (mean ± SD, n = 5). **E** Immunostaining of HSD3β1 in testes of DZN-treated and control mice. Scale bar = 100 µm. **F** Concentration of serum testosterone in DZN-treated and control mice. Values on graph are presented as the mean ± SD. **G** Expression levels of *Hsd17b3*, *Cyp17a1*, *Hsd3b1*, and *Cyp11a1* in testis from each group. Values on graph are presented as the mean ± SD (log2 scale) (n = 5). **H** Protein expression levels of HSD3β1 in testes of DZN-treated and control mice. The graph shows the relative protein levels (mean ± SD, n = 5). Asterisks indicate significant differences between the treatment and control groups at *p < 0.05, **p < 0.01, and ***p < 0.001. All statistical analyzes for the graphs were conducted using a t-test.

Based on previous results, DZN induced germ and sperm cell damage in mice testes. Alterations in Sertoli and Leydig cells in testicular sections from both the control and DZN-treated groups were examined (Fig. 2). Immunostaining of SOX9, a Sertoli cell marker, showed fewer SOX9+ cells in the DZN-treated groups than in the control group. The number of SOX9+ cells within the seminiferous tubules was reduced by 25% in the DZN-treated groups compared to the control group (Fig. 2A, B). To verify the SOX9+ cell count results, the gene and protein expression of Sertoli cell markers were measured. Expression levels of Sertoli cell markers, such as *Sox9*, Anti-Mullerian Hormone (*AMH*), and Wilms’ tumor gene1 (*WT1*) were significantly reduced in the DZN-treated group, with *Sox9*, *AMH*, and *WT1* decreasing by −1.47, −0.75, and −1.28 (log2 scales), respectively, compared with the control group (Fig. 2C). Similar patterns were observed at the protein level, where *Sox9* expression was notably reduced by 50% in the DZN-treated group compared to the control group (Fig. 2D). Regarding the effect of DZN on steroidogenesis, 3beta HSD1-positive cells were reduced within the interstitial zone of the testis of DZN-treated mice compared to that of the control group (Fig. 2E). Serum testosterone levels in DZN-treated group (1.59 ng/mL) were significantly deceased compared to that in the control group (3 ng/mL) (Fig. 2F). Additional analysis at the mRNA level unveiled significant reductions in HSD17beta3, Cyp17a1dms, and HSD3beta1, Cyp11a1 by −1.76, −0.77, −0.62, and > −2.3 (log2 scales), respectively, in the DZN-treated group compared to the control group (Fig. 2G). Consistent with mRNA levels, protein analysis indicated a notable reduction in the expression of 3beta HSD in the DZN-treated group compared to the control group (Fig. 2H).

### DZN induces the generation of ROS and cell death in testis

**Fig. 3:**
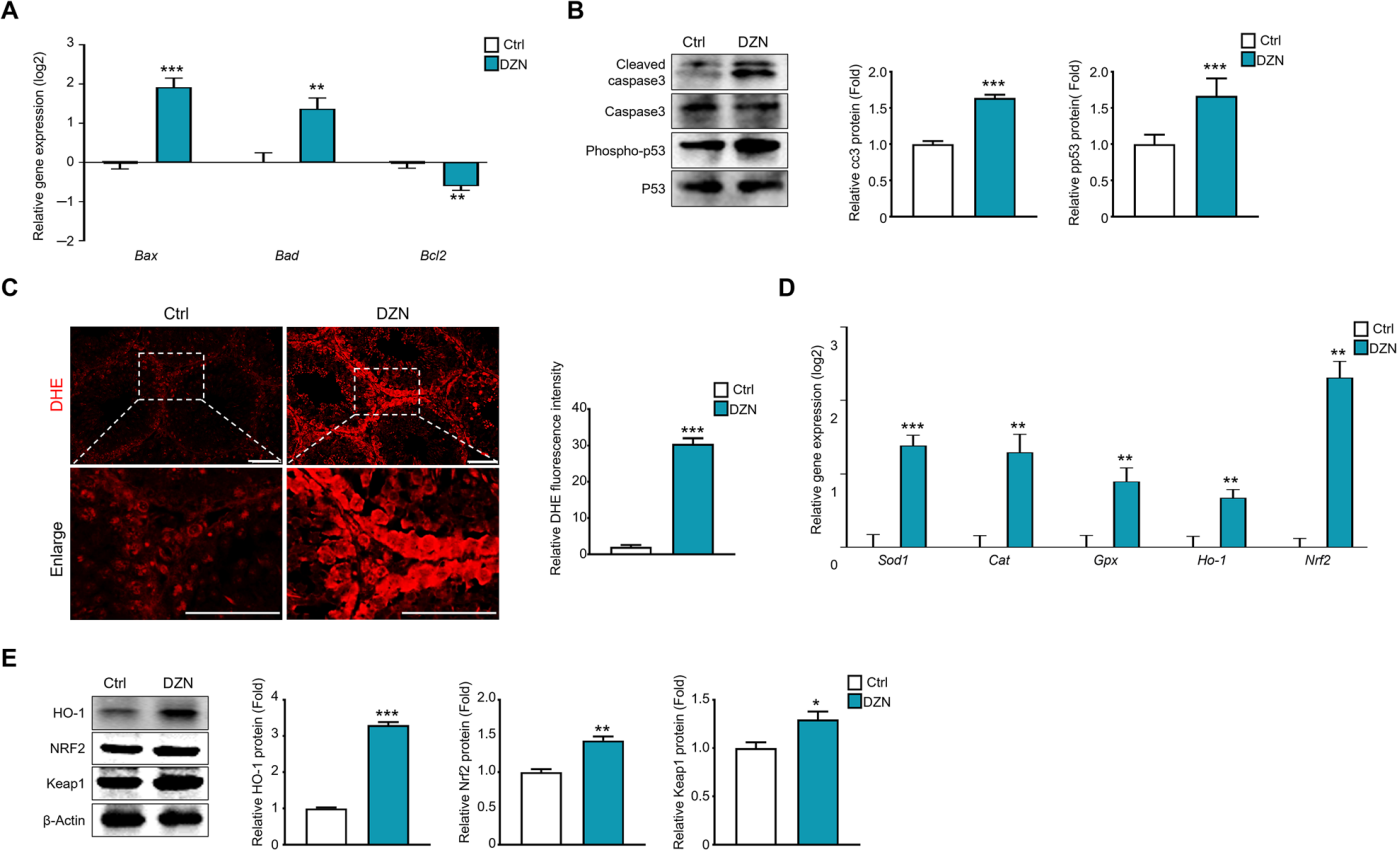
DZN leads to apoptosis via ROS generation in mice testes. **A** Expression levels of *Bax*, *Bad*, and *Bcl2* in testis from each group. values on graph are presented as the mean ± SD (log2 scale) (n = 5). **B** Protein expression levels of cleaved caspase3, caspase3, phospho-p53, and P53 in testes of each experiment group. The graph shows protein expression levels relative to inactive form (mean ± SD, n = 5). **C** DHE fluorescence staining for detecting of ROS in testes from each experiment groups. Scale bar = 50 µm. The graph indicated fluorescent intensity of ROS using Image J software. **D** Expression levels of *Sod1, Cat, Gpx, Ho-1*, and *Nrf2* in testis from each group. Data are presented as the mean ± SD (log2 scale) (n = 5). **E** Protein expression levels of HO-1, NRF2, and KEAP1. The graph shows relative protein levels (Mean ± SD, n = 5). Asterisks indicate significant differences between the treatment and control groups at *p < 0.05, **p < 0.01, and ***p < 0.001. All statistical analyzes for the graphs were conducted using a t-test.

Excessive production of reactive oxygen species (ROS) can modify and damage cellular proteins, lipids, and DNA, ultimately leading to cell death. apoptotic cell death in testis and generated oxidative stress following DZN exposure was assessed using DHE staining. At the mRNA level, Bax and Bad significantly increased by 1.9-fold and 1.4-fold (log2 scale), respectively, in the DZN-treated group compared to the control group, and Bcl2 decreased by 0.6-fold (log2 scale) (Fig. 3A).

DHE is a red fluorescent probe that is commonly used to detect ROS. When ROS are present, they oxidize DHE, converting it into ethidium, a fluorescent compound that can intercalate into cellular DNA. As shown in Fig. 3C, almost no DHE fluorescence was detected in the control group, whereas significant fluorescence was observed in the testes of the DZN-treated group (Fig. 3C). DHE-positive areas were found in both the inner and outer seminiferous tubules in testis. Additionally, in the DZN-treated group, DHE expression intensity was significantly increased by >15.2-fold compared to that in the control group. (Fig. 3C). To verify of DHE staining results, mRNA expression levels of ROS-related biological markers SOD1, CAT, GPX, HO-1, and Nrf2 were evaluated; results showed that all genes were significantly upregulated in the DZN-treated group compared to the control group (Fig. 3D). Consistently, the protein expression levels of HO-1, Nrf2, and Keap1 were also significantly increased in the DZN-treated group compared to those in the control group (Fig. 3E).

### DZN inhibits proliferation of testicular cell lines

**Fig. 4:**
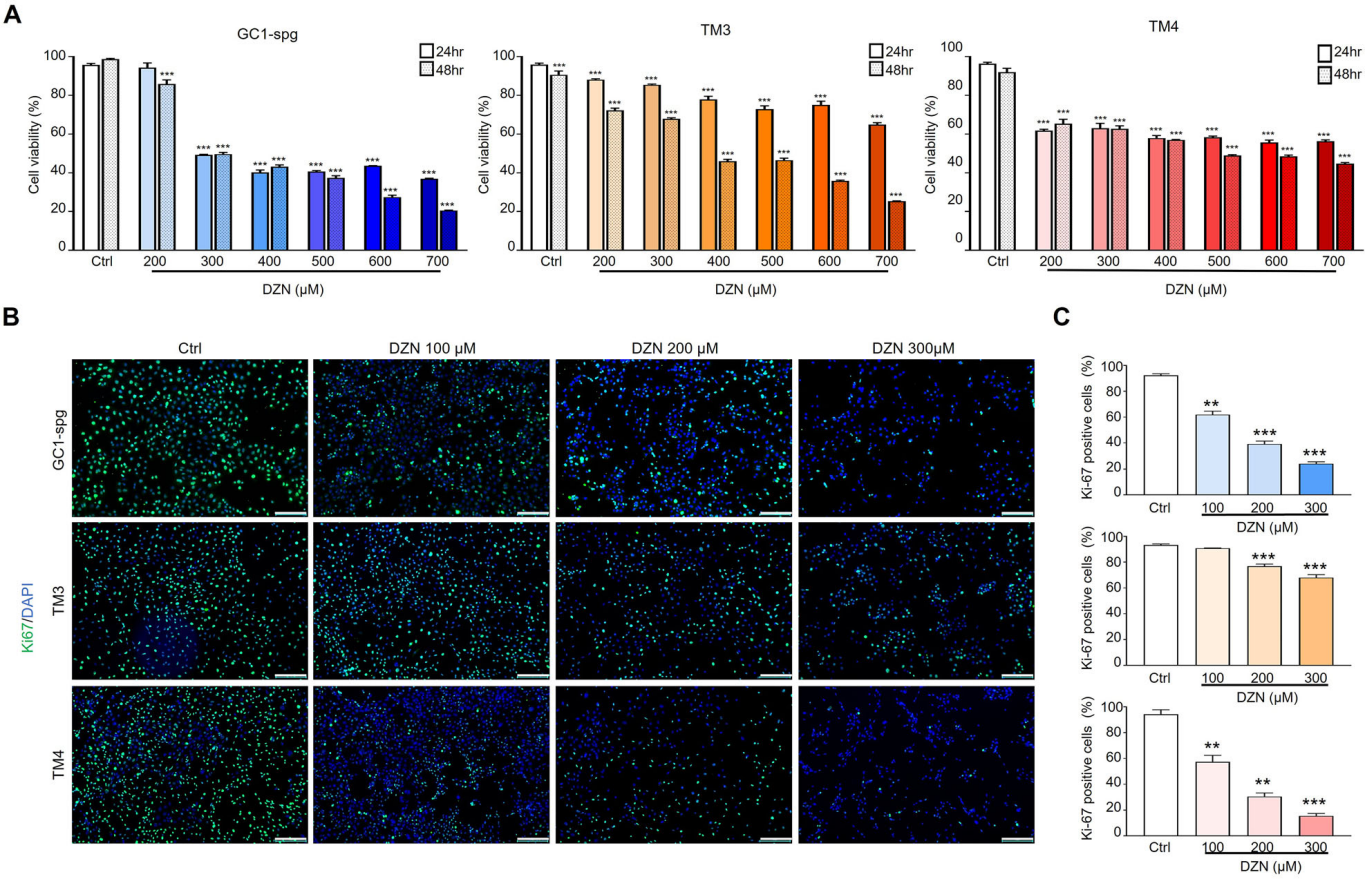
Effect of DNZ cell viability and rate of proliferation in testicular cells (GC-1 spg, TM3, and TM4). **A** Cell viability was assessed in GC1 spg, TM3, and TM4 cells following treatment with DZN (0–700 µM for 24 h and 48 h). DMSO was used as the control treatment. Values on graph are presented as the mean ± SD of three independent experiments. **B** Ki67 immunostaining of GC1 spg, TM3, and TM4 cells exposed to 0–300 µM DZN for 24 h. Scale bar = 100 µm. **C** Graph showed the percentage of Ki67 positive cell in GC-1 spg, TM3, and TM4 cells after DZN treatment at 24 h (n = 3). Asterisks indicate significant differences between the treatment and control groups at **p < 0.01 and ***p < 0.001.

Our in vivo experiment showed the negative effect of DZN in each of testicular cell types, including germ, Sertoli, and Leydig cells. Therefore, we investigated the underlying mechanism of DZN toxicity on each testicular cell line (GC-1 spermatogonia (spg), TM3 Leydig, and TM4 Sertoli cells). Cell viability assays were performed after treating the three testicular cell types with 0–700 µM DZN for 24–48 h. In GC-1 spg cells, DZN reduced cell viability to <50% at 300 µM. Similarly, TM4 cells derived from Sertoli cells showed a concentration-dependent pattern, with cell viability reduced to <65% when treated with DZN concentrations > 200 µM. TM3 cells also showed a dose-dependent decrease in cell viability when treated with DZN for 24 h. At 48 h of treatment with 200 µM DZN, the cell viability of TM3 cells decreased to <50% (Fig. 4A). The effect of DZN on testicular cell lines was further confirmed by Ki67staining, which indicates cell proliferation (Fig. 4B). At a DZN concentration of 100 µM, GC-1 spg and TM4 cells had 62% and 57% Ki67-positive cells, respectively, and TM3 cells had 68% Ki67-positive cells after exposure to 300 µM DZN (Fig. 4C). These results indicate that DZN significantly impairs cell viability and proliferation in testicular cell lines in a dose-dependent manner.

### DZN-induced apoptotic cell death of testicular cell, GC-1 spg, TM3, and TM4 cell

**Fig. 5:**
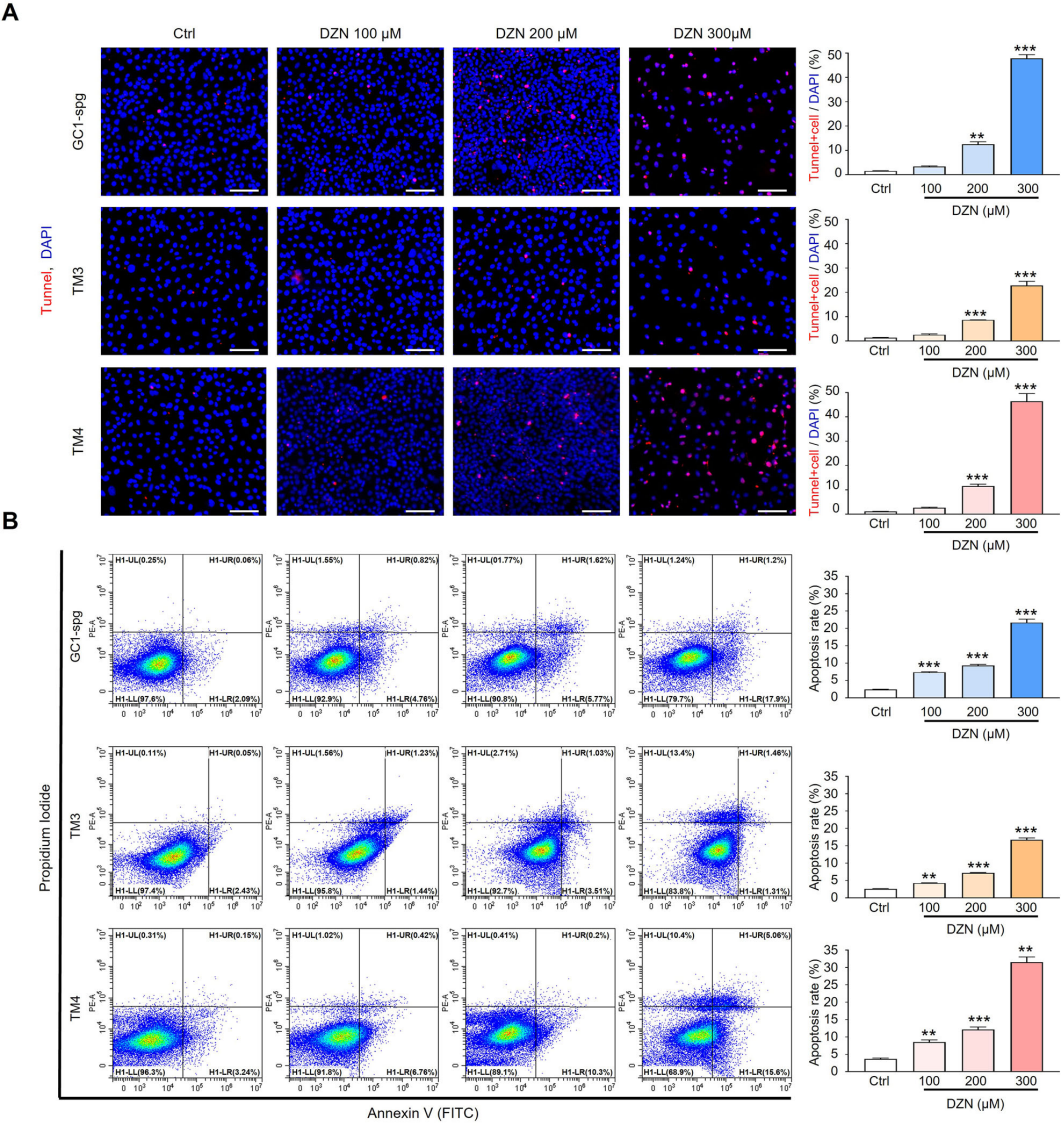
DZN induces apoptosis in testicular cells (GC-1 spg, TM3, and TM4). **A** Detection of in situ DNA fragmentation using a TUNEL assay. TUNEL-positive nuclei (red fluorescent) increased in a dose-dependent manner in DZN-treated GC-1 spg, TM3, and TM4 cells. Scale bar = 100 µm. The graphs present Tunnel/DAPI rate analysis as a percentage (mean ± SD, n = 5). **B** Representative flow cytometry plots for Annexin V (FITC) and propidium iodide (PI) stained cells. The graphs present apoptosis rate analysis as a percentage of apoptotic cells number (mean ± SD, n = 5). Asterisks indicate significant differences between the treatment and control groups at **p < 0.01 and ***p < 0.001.

As shown in Fig. 4 proliferation of testicular cells was significantly reduced after exposure to DZN. The TUNEL assay identifies DNA fragmentation. Thus, TUNEL assays were performed on testicular cells after treatment with 0–300 µM DZN. The number of TUNEL-positive cells increased in a dose-dependent manner in GC-1 spg, TM3, and TM4 cells (Fig. 5A). To verify the TUNEL assay results, flow cytometry was performed using the Dead Cell Apoptosis Kit to assess the proportion of apoptotic cells (both early and late apoptosis) in GC-1 spg, TM4, and TM3 cells exposed to 0–300 µM DZN. As shown in Fig. 5B, the rate of apoptosis in GC-1 spg, TM4, and TM3 cells after exposure to DZN at concentrations ranging from 200 to 300 µM for 24 h, was significantly increased compared to that in the control treatment. Approximately 20–30% of cells exhibited apoptosis in the group exposed to 300 µM DZN (Fig. 5B). These results indicate that DZN significantly impairs the cell viability of testicular cell lines and induces apoptosis in a dose-dependent manner.

### DZN induces ROS production by increasing mitochondrial oxidative stress in testicular cells

**Fig. 6:**
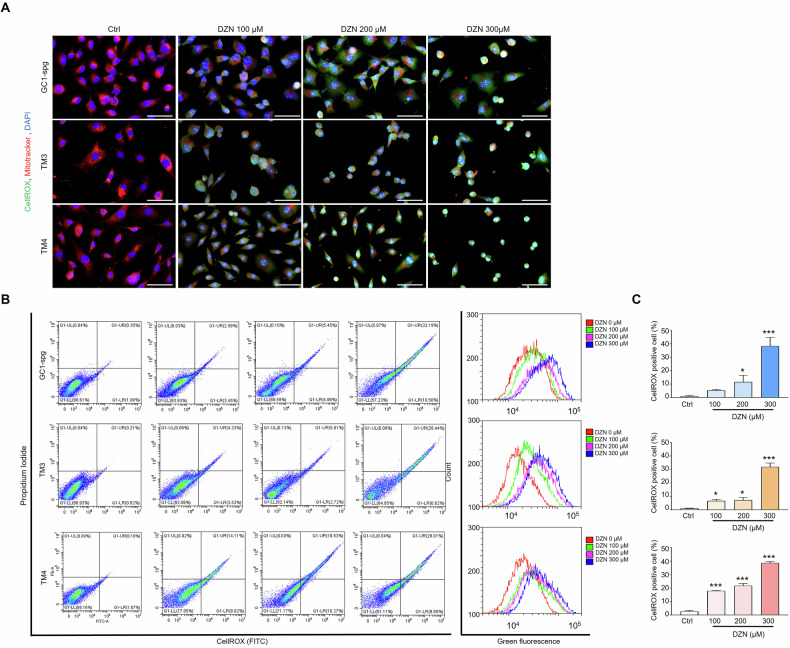
Effects of DZN on mitochondrial ROS production on testicular cells (GC-1 spg, TM3, and TM4). **A** Each cell was double stained with CellROX® FITC and MitoTracker Red to measure oxidative stress levels. CellROX® staining indicates oxidative stress, whereas the MitoTracker Red CMXRos signal indicates changes in mitochondrial membrane potential. Scale bar = 50 μm. **B** Flow cytometry dot plots for GC-1 spg, TM3, and TM4 cells stained with CellROX® for quantification. Histograms of ROS production obtained by flow cytometry after CellROX® staining of DZN-treated GC-1 spg, TM3, and TM4 cells. **C** Graph presents the quantitative change of CellROX in each group, and data are presented as the mean ± SD of five independent replicates. Asterisks indicate significant differences between the treatment and control groups at *p < 0.05 and ***p < 0.001.

Based on DHE staining results shown in Fig. 3, the production of intracellular ROS in GC-1 spg, TM3, and TM4 cells after treatment with DZN was investigated (Fig. 6). Results showed that exposure to 100 µM DZN triggered ROS production in the mitochondria of all three testicular cell types, as indicated by visually elevated CellROX® FITC signals in DNZ-treated cells compared to the untreated control cells (Fig. 6A). For quantification of ROS production, flow cytometry was performed after CellROX® FITC labeling in each different cell and showed that exposure to 100–200 µM DZN distinctly increased ROS production in each cell type in a dose-dependent manner (Fig. 6B, C), which is consistent with our in vivo data.

### Effects of DZN on the gene and protein expression of apoptosis-and ROS-related factors

**Fig. 7:**
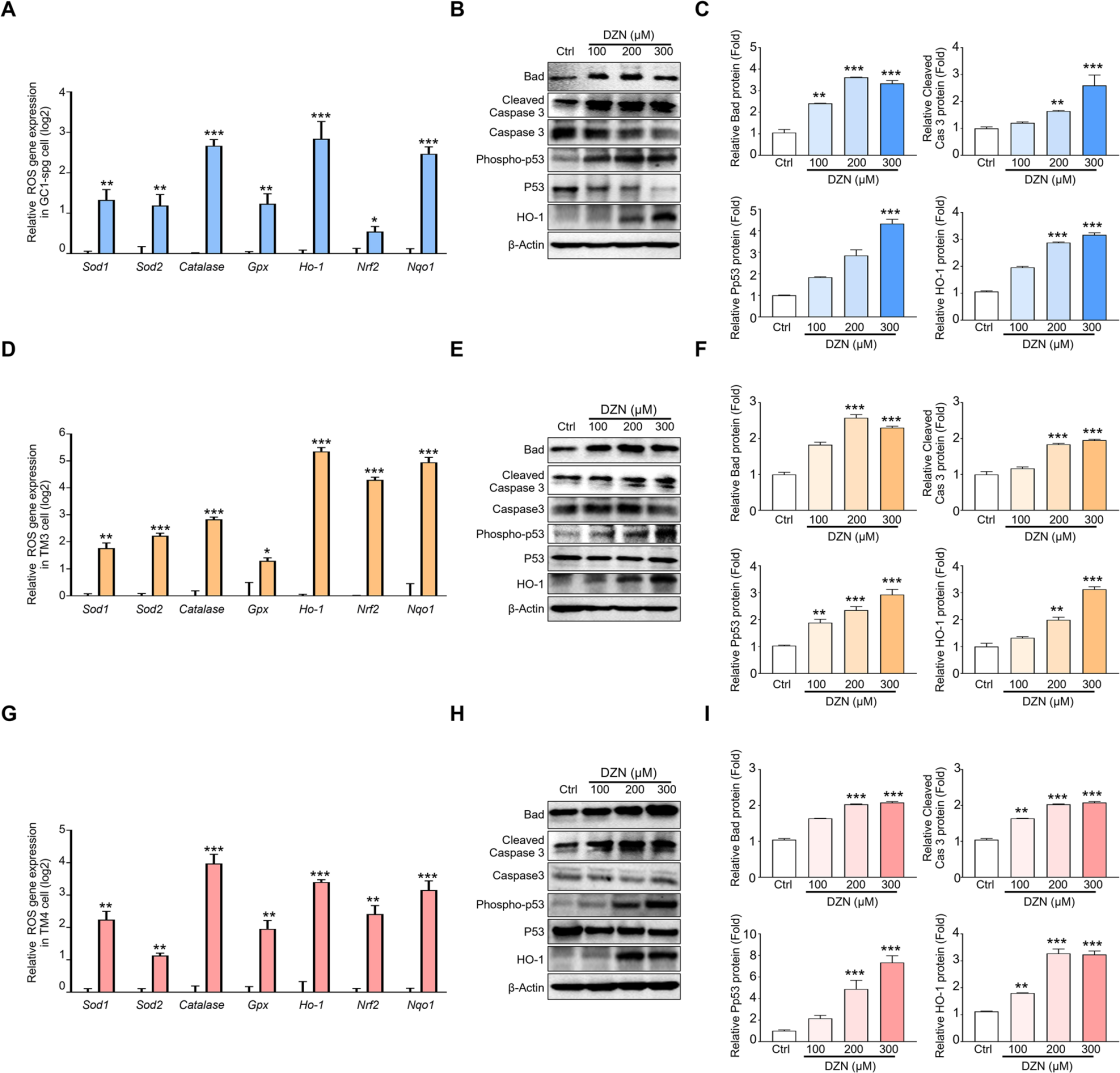
Expression of ROS and apoptotic marker in DZN exposed testicular cells (GC-1 spg, TM3, and TM4). **A** *Sod1, Sod2, Cat, Gpx, Ho-1, Nrf2*, and *Nqo1* expression in GC1-spg cells treated with 300 µM DZN for 24 h. Values on graph are presented as the mean ± SD (log2 scale) (n = 5). **B** Protein expression levels of Bad, Cleaved-caspase 3, Caspase 3, Phospho-p53, P53, and HO-1 in GC1-spg cells exposed to 0–300 µM DZN. **C** Graph shows relative protein levels (mean ± SD, n = 5). **D** *Sod1*, *Sod2*, *Cat*, *Gpx*, *Ho-1*, *Nrf2*, and *Nqo1* expression in TM3 cells before and after exposure to 300 µM DZN. Values on graph are presented as the mean ± SD (log2 scale). **E** Protein expression levels of Bad, Cleaved-caspase 3, Caspase 3, Phospho-p53, P53, and HO-1 in TM3 cells exposed to 0–300 µM DZN. **F** Graph shows relative protein levels (mean ± SD, n = 5). **G** *Sod1*, *Sod2*, *Cat*, *Gpx*, *Ho-1*, *Nrf2*, and *Nqo1* expression in TM4 cells before and after exposure to DZN. Values on graph are presented as the mean ± SD (log2 scale). **H** Protein expression levels of Bad, Cleaved-caspase 3, Caspase 3, Phospho-p53, P53 and HO-1 in TM4 cells exposed to 0, 100, 200 and 300 µM DZN. **I** Graph shows relative protein levels (mean ± SD, n = 5). Asterisks indicate significant differences between the treatment and control groups at *p < 0.05, ** p < 0.01, and ***p < 0.001.

To confirm that ROS production increased in DZN-treated testicular cells, gene and protein expression levels of apoptosis-related factors, such as Bad, Cleaved-Caspase 3, Phospho-p53 and ROS-related factors, such as *Sod1, Sod2, Catalase, Gpx, Ho-1, Nrf2*, and *Nqo1* were measured in each testicular cell type (Fig. 7). The mRNA expression levels of *Sod1, Sod2, Catalase, Gpx, Ho-1, Nrf2*, and *Nqo1* were significantly increased in all cell types after treatment with 300 μM DZN compared to that in the control group, and the mRNA expression levels of all the relevant genes were significantly increased in DZN-treated GC-1 spg cells compared to that in the control GC-1 spg cells; particularly, *Catalase*, *Ho-1*, and *Nqo-1* increased more than 4-fold compared to the control group (Fig. 7A). Protein expression levels of BAD, Cleaved-Caspase 3, Phospho-p53, and HO-1 were also clearly increased in dose-dependent manner in DZN-treated samples. Specifically, BAD, Cleaved Caspase 3, Phospho-p53, and HO-1 increased by 3.34-, 2.6-, 4.33-, and 3.17-fold, respectively (Fig. 7B, C).

In TM3 Leydig cells, *Sod1, Sod2, Catalase, Gpx, Ho-1, Nrf2*, and *Nqo1* expression increased significantly after DZN treatment. Notably, *Ho-1*, *Nrf2*, and *Nqo1* expression was 16-fold higher in the DZN-treated group compared to that in the control group (Fig. 7D). The protein expression of BAD, Cleaved-Caspase 3, Phospho-p53, and HO-1 increased by 2.3-, 1.95-, 2.92-, and 3.12-fold, respectively (Fig. 7E, F).

In TM4 Sertoli cells, *Sod1, Sod2, Catalase, Gpx, Ho-1, Nrf2*, and *Nqo1* expression was higher in the DZN-treated cells compared to the control cells, with *Catalase*, *Ho-1*, and *Nqo1* expression increasing by more than 8-fold (Fig. 7G). The protein expression levels of BAD, Cleaved-Caspase 3, Phospho-p53, and HO-1 increased by 2.9-, 2.08-, 7.34-, and 3.23-fold, respectively, in the DZN-treated groups compared to that in the control group (Fig. 7H, I). This result indicated that the ROS- and apoptosis-related factors increased in all three testicular cell types after DZN exposure.

### ROS inhibitor N-acetyl-l-cysteine (NAC) suppresses DZN-induced apoptosis in testicular cells

**Fig. 8:**
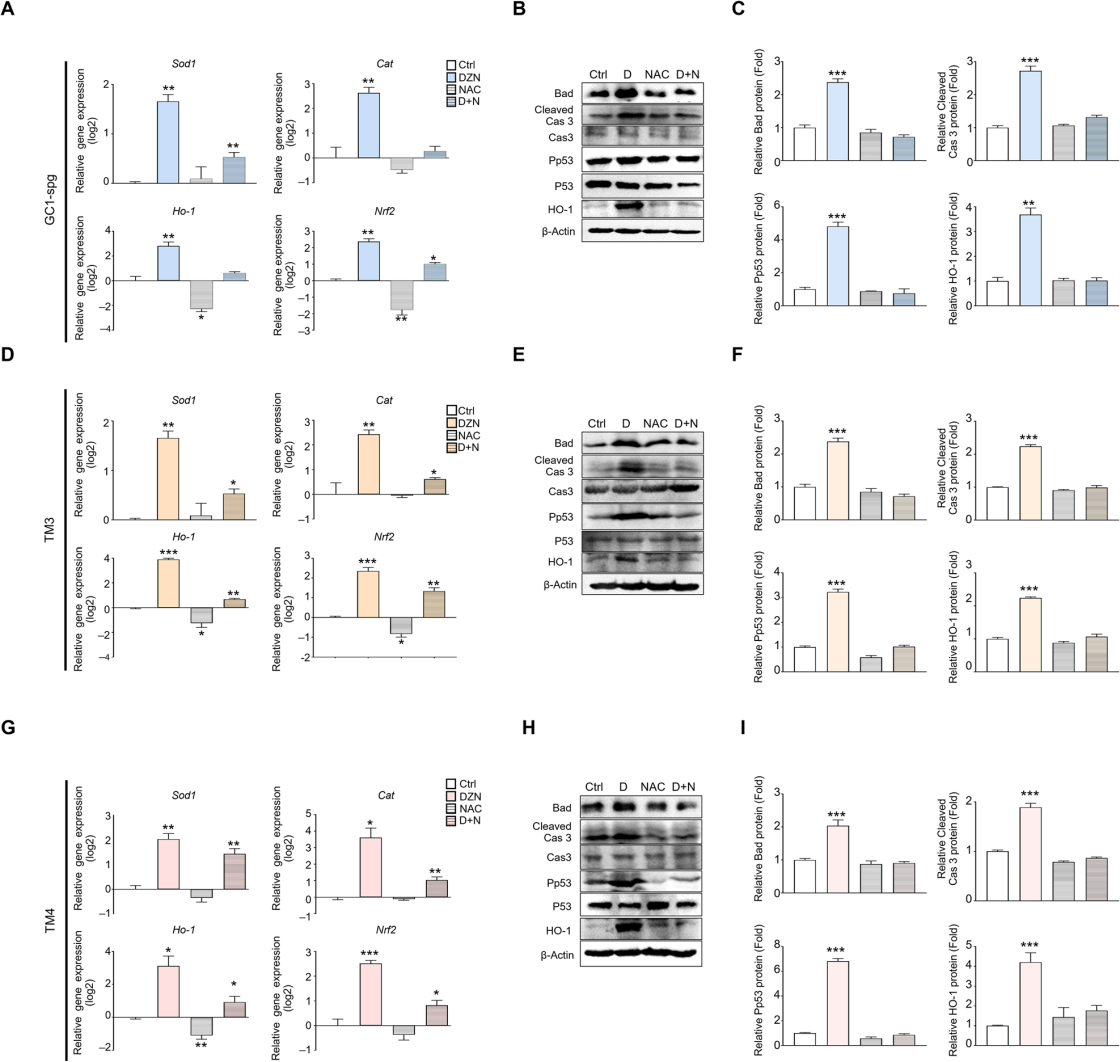
NAC suppresses expression of pro-apoptotic and ROS marker in DZN treated testicular cells (GC-1 spg, TM3, and TM4). **A** *Sod1*, *Cat*, *Ho-1*, and *Nrf2* expression in GC-1 spg cells for each treatment (control, DZN, NAC, and DZN + NAC). Values on graph are presented as the mean ± SD (log2 scale). **B** Protein expression levels of Bad, Cleaved-caspase 3, Caspase 3, Phospho-p53, P53, and HO-1 in GC-1 spg cells for each treatment. **C** Graph shows relative protein levels (mean ± SD, n = 5). **D** *Sod1*, *Cat*, *Ho-1*, and *Nrf2* expression in TM3 cells for each treatment. Values on graph are presented as the mean ± SD (log2 scale). **E** Protein expression levels of Bad, Cleaved-caspase 3, Caspase 3, Phospho-p53, P53, and HO-1 in TM3 cells. **F** Graph shows relative protein levels (mean ± SD, n = 5). **G** *Sod1*, *Cat*, *Ho-1*, and *Nrf2* expression in TM4 cells for each treatment. Values on graph are presented as the mean ± SD (log2 scale). **H** Protein expression levels of Bad, Cleaved-caspase 3, Caspase 3, Phospho-p53, P53, and HO-1 in TM4 cells for each treatment (control, DZN, NAC, and D + N). **I** Graph shows relative protein levels (mean ± SD, n = 5). Asterisks indicate significant differences at *p < 0.05, ** p < 0.01, and *** p < 0.001.

To clarify whether apoptosis is regulated by ROS production in DZN-treated samples. NAC was used as an ROS inhibitor to confirm that DNZ blocks ROS generation and reduces cell death. In GC-1 spg cells, the expression of ROS-related genes such as *Sod1, Catalase, Ho-1*, and *Nrf2* were upregulated after treatment with 300 μM DZN. However, in the group treated with both NAC (5 mM) and DZN simultaneously, the expression of these genes was lower compared to that in the DZN-only treatment (Fig. 8A). The protein expression levels of Bad, Cleaved-caspase 3, Phopho-p53, and HO-1, which are associated with apoptosis and ROS production were upregulated after treatment with DZN; the elevated protein expression after DZN treatment was suppressed by NAC (Fig. 8B, C). Similarly, in TM3 cells, *Sod1, Catalase, Ho-1*, and *Nrf2* expression was upregulated after DZN treatment, but suppressed by NAC treatment (Fig. 8D). Protein levels of Bad, Cleaved-caspase 3, Phospho-p53, and HO-1 were higher in the DZN-treated groups compared to that in the control group, but remained the same in the NAC + DZN-treated group (Fig. 8E, F). In TM4 cells, *Sod1, Catalase, Ho-1*, and *Nrf2* expression was upregulated, but this upregulation was suppressed by NAC treatment (Fig. 8G). Expression of apoptotic proteins, BAD, Cleaved-caspase 3, and Phospho-p53 increased after DZN treatment, but this increase was suppressed by NAC treatment (Fig. 8H, I).

## Discussion

Testicular pathology (cryptorchidism, testicular torsion, and testicular cancer), lifestyle (drinking, smoking, and diet), energy dyshomeostasis, metabolic disorders, pharmaceuticals, and environmental toxicants (plasticizers, toxins, organic chemicals, and pesticides) can affect to male reproductive function. Particularly sperm production and motility [24–26]. DZN, an OP insecticide commonly used to control household insects and protect fruit and vegetable crops, has been associated with serious health issues in several laboratory animals [4].

Our result demonstrated that mice treated with DZN exhibited a body weight reduction exceeding 4 g compared to control mice. Metabolic disturbances caused by Ops exposure may enhance lipolysis and protein catabolism, with the consequent weight loss potentially exacerbating metabolic stress due to nutrient deficiency [27]. Additionally, anorexia-induced reductions in food and water intake may elevate leptin levels, subsequently contributing to testosterone reduction associated with spermatogenesis in male mice [28].

The current study investigated the mechanism by which DZN increases testicular damage and sperm malformations in mice through the administration of 30 mg/kg DZN injections for 11 weeks. In vivo, no significant difference was observed in the testis weight of DZN-exposed ICR mice compared to the control group; however, the diameter of seminiferous tubules was significantly decreased. Testicular weight and seminiferous tubule diameter are important indicators of overall testicular health as they reflect germ cell differentiation, loss, and other changes [29]. The seminiferous tubules are specialized structures responsible for sperm production, undergoing continuous rearrangement throughout male development reproduction [30]. In these biological structures, spermatozoa are the products of the germ cell lineage, created through spermatogenesis, a complicated developmental process that occurs through interaction between germ and somatic cells [31]. Abnormalities in seminiferous tubule diameter may influence the internal environment of the tubules, and as anticipated, this change decreased the number of germ and Sertoli cells per tubule. In particular, both undifferentiated and meiotic germ cells were reduced in testes of the DZN-treated mice. Meiotic germ cells play a crucial role in spermatogenesis because they undergo meiosis [32]. Although they did not use a rodent model, one study described DZN-induced reproductive toxicity and meiotic dysfunction in the roundworm *Caenorhabditis elegans* [33].

The CASA-based analysis results showed that DZN has a negative effect on sperm parameters in the testis; an increase of > 60% in the number of sperm with an abnormal neck shape was observed in the DZN-treated group compared to the control group. Similarly, Sarabia et al. reported that DZN caused sperm deformities by damaging testicular tissue and altering DNA or associated proteins in mice [34]. In humans, the sperm count, sperm concentration, progressive motility, total motility, and normal morphology in individuals exposed to OP pesticides decreased compared to those who were not exposed in a sample of 766 male subjects (349 exposed to OP pesticides, 417 unexposed [control]) [35].

Dutta et al. studied the effects of DZN on the testes of bluegill (*Lepomis macrochirus*), revealing a disrupted testis structure and fluctuating seminiferous lumen diameters over time. After 24 h, sperm cells enlarged while spermatids shrank. By 48 h tubules became denser, and by 72 h, they dilated. At 96 h up to 2 weeks, the structure became loose and dense with minimal lumen visibility. Irregular diameter changes likely stem from weakened connective tissue due to DZN exposure, potentially hindering sperm production and threatening population dynamics. No clear correlation was found between fish size, body weight, and testis weight [36]. Another study reported germ cell sloughing, necrosis, and damage to seminiferous tubules in 4-week-old rats, which led to a Sertoli cell-only phenotype when exposed to DZN at sub-chronic doses for 90 days via drinking water [37].

In the present study, DZN was confirmed to affect germ and Sertoli cells and steroidogenesis in mice testes. The number of Sertoli cells, the expression of steroidogenesis-related markers, and serum testosterone levels were decreased in the testes of DZN-treated mice. Mighani et al. investigated the impact of DZN on gene expression related to spermatogenesis (activin, androgen receptor, and FSH receptor) and sex hormones in male zebrafish exposed to different DZN concentrations (5, 10, and 20% LC_50_) for one month. They showed a significant decrease in the expression of FSH receptor, activin, and androgen receptor genes as the DZN concentration increased. Sex hormone levels also decreased, except for estradiol, which increased with higher DZN levels. Overall, DZN exposure may disrupt reproductive efficiency in zebrafish. [38]. In a previous study using a female rat model, the impact of DZN on corpus luteum (CL) function was investigated. DZN was administered after hormonal stimulation with PMSG and hCG, and it significantly altered the expression of *StAR*, an important gene in steroidogenesis, in a time-dependent manner and reduced CL diameter compared to that in the control group. This indicates that DZN disrupts CL function and may contribute to female reproductive damage [39].

According to our in vivo experiment results, the effects of DZN on testes and male reproductive function are not cell-specific and may affect various testicular cells. Therefore, we investigated the effect of DZN on various testicular cells. Furthermore, ROS production and expression of apoptotic markers were clearly increased in the testes of DZN-treated mice. Many studies have reported an increase in ROS generation after DZN exposure. Jafari et al. reported that DZN induced oxidative stress in the brain, heart, and spleen of Wistar and Norway rats, with Wistar rats being more sensitive. Higher DZN doses increased oxidative stress markers and altered antioxidant enzyme activities, leading to significant tissue damage, particularly in the brain [40]. Another study evaluated the nephrotoxic effect of DZN in rats and found that it significantly increased renal lipid peroxidation, decreased antioxidant enzyme activities, and reduced glutathione levels, indicating its ROS-generation potential [6]. Oskay et al. reported that DZN induced oxidative stress in rat testes, indicated by increased lipid peroxidation and decreased GSH, Vitamin C and E, and β-carotene levels, which could be mitigated by NAC treatment, which reduced lipid peroxidation and boosted antioxidant levels [41]. However, this study did not provide a detailed analysis of testicular damage; it only measured the levels of ROS indicators, such as GSH-Px and lipid peroxidation in testes [41].

Mitochondria serve as the primary energy-generating organelles within cells and are well-established targets of toxicity induced by various drugs and xenobiotics [42]. Several studies in murine models have demonstrated that DZN disrupts the activity of antioxidant enzymes, particularly glutathione peroxidase, which plays a critical role in renal metabolism [43]. Furthermore, in mice liver, mitochondrial dysfunction is closely linked to oxidative stress, as the leakage of electrons from the electron transport chain renders mitochondria the principal source of reactive oxygen species (ROS) generation [44, 45]. Excessive ROS production can trigger the opening of the mitochondrial permeability transition pore, leading to mitochondrial depolarization, uncoupling of oxidative phosphorylation, and ultimately, membrane rupture [46, 47]. In addition, studies on murine hepatic mitochondria have shown that DZN inhibits state III respiration, which is driven by glutamate and malate, substrates of complex I of the respiratory chain, as well as succinate, a substrate of complex II [10]. This inhibition is associated with a reduction in mitochondrial membrane potential, suppression of ATP synthesis, and oxidation of endogenous NAD(P)H and protein thiol groups. Additionally, DZN has been reported to induce mitochondrial permeability transition through a mechanism sensitive to cyclosporin A, EGTA, ruthenium red, and N-ethylmaleimide, potentially leading to mitochondrial Ca^2+^ efflux and cytochrome c release triggered by ROS [10].

In the present study, DHE staining results of the testes from DZN-treated mice showed that ROS production is not cell-type specific, with DHE-stained cells observed in both the inner and outer seminiferous tubules. Based on this result, the effects of DZN on three different testicular cell types were investigated to reveal the mechanisms underlying the adverse effects of DZN exposure. Although some studies reported that DZN induces ROS production in various cells, no studies have investigated whether the effects of DZN on testicular cells, such as germ, Sertoli, and Leydig cells, are similar in the different cell types. In the present study, our in vitro experiments confirmed that DZN induced ROS generation and apoptosis equally in all three cell types and that apoptosis was inhibited by NAC, an ROS inhibitor. Our results clearly showed that the expression of oxidative stress and apoptosis-related markers in all three cell types were increased after DZN treatment but suppressed when simultaneously treated with NAC.

A previous study reported the effect of DZN on cell proliferation and apoptosis in germ cells in rat testes in vivo. Their results demonstrated that DZN induced testicular toxicity by decreasing proliferation and increasing apoptosis in testicular germ cells [48]. In fish, reduction of germ cell size (spermatogonia) was observed in DZN-exposed bluegill for 2 weeks [36]. Effects of other substances in OP-based insecticides, such as chlorpyrifos (CPF), have also been reported. Chen et al. reported that CPF induces cell death and apoptosis in testicular cells, including GC-1 spg, TM3, and TM4 cells via oxidative stress in via increased p-AMPK levels [49]. Although not specifically in the testes, many studies have reported that increased ROS generation caused by OPs contributes to the toxicity of various pesticides [50]. The results of these studies support our in vivo and in vitro results.

## Conclusions

Our findings revealed clear in vitro and in vivo effects of DZN on male reproductive organs and three different testicular cell types (GC-1 spg, Sertoli, and Leydig cells). In vivo, DZN administration decreased normal sperm parameters related to motility and survivability and reduced the number of spermatogonia, Sertoli cells, and steroidogenesis by regulating the expression of markers related to male hormone synthesis. In addition, pro-apoptotic- and ROS-related factors were significantly increased in mice testes after DZN treatment. In vitro, DZN treatment induced ROS-mediated cell apoptosis in all three testicular cell types. Our findings suggest that DZN has similar effects on the various testicular cell types and does not target specific cell types. Our detailed report on the effects of DZN using in vivo and in vitro models provides essential data for studies on environmental male reproductive toxicity, subfertility, and infertility. Furthermore, to our knowledge, there have been no reports on the effects of DZN on GC-1 spg, TM3 Leydig, and TM4 Sertoli cells, making our report the first.

## Methods

### Experimental animals

Six-week-old ICR male mice were purchased from Dae Han Bio Link Co. (Daejeon, Korea). Mice were maintained in a controlled environment: temperature range, 20–26 °C; relative humidity, 40–60%; and a 12 h/12 h light-dark cycle. There were ten mice distributed to each group for experiments randomly. DZN was diluted in corn oil, and the mice were administered 30 mg/kg/day of DZN via intraperitoneal injection (100 μL) for 11 weeks. Body weight was measured once per week, and testis weight was measured on the last day of the experimental period. Blood was collected by cardiac puncture on the last day of the experiment. All experiments using animals strictly adhered to the guidelines provided by the Institutional Animal Care and Use Committee (IACUC) of Sangji University (approval protocol #2023-9).

### Sperm collection and parameters and testosterone measurement

Mice were sacrificed at the end of the experimental period. Spermatozoa were collected from the cauda epididymis and incubated in M2 medium for 60 min at 37 °C. Further, 20 µL aliquot of sperm was placed in a chamber to evaluate sperm motility parameters using computer-assisted sperm analysis (CASA). All procedures were performed following a previously described method [51, 52]

A sperm pellet was smeared on a glass slide for teratozoospermia analysis and stained with Diff-Quick staining solution (Megascience, Seoul, Korea) according to the manufacturer’s instructions. The sperm morphology was observed using a microscope (Olympus, CKX53, Japan). Serum testosterone levels were measured using a testosterone enzyme-linked immunosorbent assay (ELISA) kit (Cusabio biotech, Houston, TX, USA) following the manufacturer’s instructions.

### Testicular cell line culture and treatment

Three testicular cell lines, GC-1 spermatogonia (spg), TM3 Leydig, and TM4 Sertoli cells, were purchased from the Korea Cell Bank (KCLB, Seoul, South Korea). The cells were cultured in Dulbecco’s Modified Eagles Medium (DMEM; 10% fetal bovine serum, 1% penicillin-streptomycin antibiotics) at 37 °C in an incubator with 5% CO_2_ atmosphere. DZN (Sigma Aldrich, St. Louis, MO, USA) stock solution (1 M) was prepared and diluted in dimethyl sulfoxide (DMSO) for further experiments.

### Cell proliferation assay

The viability of GC-1 spg, TM3 Leydig, and TM4 Sertoli cells was measured using an MTT assay EZ-Cytox kit (Daeil Lab Services Co., Seoul, Korea) according to the manufacturer’s instructions Each cell was seeded in 24-well plates at a density of 0.5 × 10^5^ cells/well and incubated at 37 °C for 12 h. The medium was then replaced with fresh medium containing DZN (0–300 μM) and further cultured for 24 h. Absorbance was measured at 490 nm using a microplate reader (Epoch, Bio Teck, Winooski, VT, USA).

### Histology and Immunostaining

For cell staining, the testicular cells were seeded on 18 mm glass coverslips in a 6-well plate (BD Biosciences, Franklin Lakes, NJ) and treated with 0–300 µM DZN for 24 h with in vitro culture. Subsequently, the cells were fixed with 4% paraformaldehyde at 16 °C for 15 min, permeabilized in phosphate buffered saline (PBS) with 0.1% Triton X-100 for 5 min at 16 °C, and incubated with first antibodies at 4 °C overnight, followed by incubation with FITC-conjugated secondary antibodies at 16 °C for 1 h. Cell images were obtained using fluorescence microscopy (IX73 Olympus, Tokyo, Japan). The cell proliferation index was calculated using the following formula: (number of Ki-67 positive cells)/(number of total cells) in each field. To achieve dual labeling of mitochondria and CellROX, after incubating GC-1 spg cells with DZN for 24 h, CellROX reagent (5 µM) was added to cells and cultured for 30 min at 37 °C. Subsequently, the samples were exposed to 500 nM MitoTracker Red CMXRos dye (Life Technologies, Carlsbad, CA, USA) and mounted with DAPI (Vector Laboratories Inc, Burlingame, CA, USA).

Histological analysis and immunostaining of mouse testis were performed following a previously described method [52]. Briefly, tissue samples were fixed in 4% paraformaldehyde for 16 h at 4 °C. Samples were then dehydrated, inserted in a paraffin block, and cut into 5 µm slices. Next, hematoxylin and eosin (H&E) staining was performed, and images were captured using a microscope (Olympus IX73, Tokyo, Japan).

Immunostaining of testes was performed as previously described [53]. The list of antibodies used is presented in Table 1. Fluorescence images were captured using an IX73 microscope (Olympus, Tokyo, Japan). Quantification analyzes were performed for five replicates, and at least 40 testes cross sections were scored (five sections per group).

**Table 1:** Primary antibodies used for immunostaining and blotting.

Antibody	Company	Catalogue number	Diluted
DDX4	Abcam	ab13840	1:300
SYCP3	Abcam	Ab97672	1:300
SOX9(IHC)	Abcam	Ab76997	1:300
SOX9(Western blot)	Abcam	Ab185966	1:300
3βHSD1	Santa Cruz Biotech	sc-515120	1:100
Bad	Cell signaling	#9239	1:1000
Cleaved-Caspase-3	Cell signaling	#9664	1:1000
Caspase-3	Cell signaling	#9662	1:1000
Phospho-p53	Cell signaling	#9284	1:1000
P53	Cell signaling	#2524	1:1000
HO-1	Santa Cruz Biotech	sc-136960	1:100
NRF2	Santa Cruz Biotech	sc-365949	1:100
Keap1	Santa Cruz Biotech	sc-514914	1:100
β-actin	Santa Cruz Biotech	sc-47778	1:100

### Dihydroethidium (DHE) staining

DHE (Sigma Aldrich, St. Louis, MO, USA) was used as a red fluorescent probe for the detection of ROS generation in mice testes. The deparaffined testes slides were incubated with 10 µM DHE for 1 h at room temperature. The slides were then washed three times with PBS for 5 min each. Images were captured using the IX73 microscope.

### Apoptosis assay

Apoptosis was evaluated via DNA fragmentation using the terminal deoxynucleotidyl transferase-mediated dUTP nick end labeling (TUNEL) assay following a previously described protocol [54]. The GC-1 spg, TM3, and TM4 cells (3 × 10^5^ cells/well) were cultured on glass slides in 6-well plates for 12 h. The cells were treated with four different concentrations of DZN (0–300 µM) for 24 h. After three washes with DPBS for 5 min, the cells were fixed in 10% neutral buffered formalin for 50 min at 24 °C, followed by three washes with DPBS. The glass slides were stained according to the manufacturer’s instructions of the in situ TMR red cell death detection kit (Roche, Mannheim, Germany).

### Flow cytometry

Flow cytometry (CytoFLEX, Beckman Coulter, Inc., Miami, FL, USA) was used to measure apoptosis and ROS levels in testicular cells following a previously described method [54]. Annexin V-fluorescein isothiocyanate (FITC) and propidium iodide (PI) staining was used to detect apoptotic cells, and CellROX green was used to detect ROS generation. After DZN treatment for 24 h, cells were harvested and washed with DPBS. After incubating GC-1 spg, TM3, and TM4 cells with DZN for 24 h, the cells were harvested, and fluorescence was detected using flow cytometry.

### RNA extraction and quantitative real-time PCR (qPCR)

Total RNA of testes and testicular cells were extracted using the RNeasy Mini Kit (Qiagen, Hilden, Germany). Then, cDNA was synthesized using the RevertAid cDNA synthesis kit (Thermo Scientific, Rockford, IL, USA) according to the manufacturer’s instructions. Quantitative PCR (qPCR) was performed using the QuantStudio 1 system (Applied Biosystems, Foster City, CA, USA), and PCR and data analysis were performed following previously described methods [55, 56]. The primers used in this experiment are listed in Table 2.

**Table 2:** Primers used for reverse transcription-polymerase chain reaction with mouse cDNA.

*Gene*	Forward primer	Reverse primer
*Gapdh*	5′-GTCGGTGTGAACGGATTTG-3′	5′-CTTGCCGTGGGTAGAGTCAT-3′
*Sall4*	5′-TCTCAGCAAGTGTCCGTGTC-3′	5′-GCATGAGGTAGCTTGGCTTG-3′
*Plzf*	5′-CCACCTTCGCTCACATACAG-3′	5′-TTGCCACAGCCATTACACTC-3′
*Piwil*	5′-TGGTGATTGGAATGGATGTG-3′	5′-ATGTGGCATCTGGAACACC-3′
*Sycp3*	5′-CAGATGCTTCGAGGGTGTG-3′	5′-AAGGTGGCTTCCCAGATTTC-3′
*Dmc1*	5′-CATTGGTGGACACATTCTGG-3′	5′-ATCCCTCCAGCGGTTATTG-3′
*Rec8*	5′-CTATCTCTCCGCCCAGCTTC-3′	5′-AGCCTCCTCCATATCAATGC-3′
*PGK2*	5′-ATCGGGCTCACAGTTCTACG-3′	5′-CACCAAGGATAGCCAGGAAG-3′
*TP1*	5′-AAATACCGGAAGAGCGTCCT-3′	5′-TTCGTCACGACTGGCATTTA-3′
*Sox9*	5′-AGTACCCGCATCTGCACAAC-3′	5′-TACTTGTAATCGGGGTGGTCT-3′
*AMH*	5′-GGGCCTCATCTTAACCCTTC-3′	5′-AGTCATCCGCGTGAAACAG-3′
*WT1*	5′-ATCCCAGGCAGGAAAGTGTG-3′	5′-GTGCTGTCTTGGAAGTCGGA-3′
*Hsd17b3*	5′-GCTCAACGATTCCTCCTGAC-3′	5′-CCACCCAACCCTAACTCTACC-3′
*Cyp17a1*	5′-TCCAGCATTGGAGAGTTTGC-3′	5′-ATGAGATGGCTTCCTGTTGG-3′
*Hsd3b1*	5′-AATCTGAAAGGTACCCAGAA-3′	5′-TCATCATAGCTTTGGTGAGG-3′
*Cyp11a1*	5′-GACAATGGTTGGCTAAACCTG-3′	5′-GGGTCCACGATGTAAACTGAC-3′
*Bax*	5′-GCTGACATGTTTGCTGATGG-3′	5′-GATCAGCTCGGGCACTTTAG-3′
*Bad*	5′-GCCCTAGGCTTGAGGAAGTC-3′	5′-GGCTCAAACTCTGGGATCTG-3′
*Bcl2*	5′-GGAAGGTAGTGTGTGTGG-3′	5′-ACTCCACTCTCTGGGTTCTTGG-3′
*Sod1*	5′-CCATCCACTTCGAGCAGAAG-3′	5′-CATACTGATGGACGTGGAACCCAT-3′
*Cat*	5′-GCAGATACCTGTGAACTGTC-3′	5′-GTAGAATGTCCGCACCTGAG-3′
*Gpx*	5′-TTCGGACACCAGGAGAATGG-3′	5′-TAAAGAGCGGGTGAGCCTTC-3′
*Ho-1*	5′-ACAAGCAGAACCCAGTCTAT-3′	5′-AGGTAGCGGGTATATGCGTGGGCC-3′
*Nrf2*	5′-TCTCCTCGCTGGAAAAAGAA-3′	5′-AATGTGCTGGCTGTGCTTTA-3′
*Sod2*	5′-CAGACCTGCCTTACGACTATG-3′	5′-CTCGGTGGCGTTGAGATTGTT-3′
*Nqo1*	5′-CTTTAGGGTCGTCTTGGC-3′	5′-CAATCAGGGCTCTTCTCG-3′

### Immunoblotting

Tissue and cell lysates were extracted from cells using RIPA buffer with protease inhibitor (Thermo Fisher Scientific, MA, USA). Total protein (30–40 μg) was loaded in 10% and 4–20% acrylamide gel (Bio-Rad, Hercules, CA, USA) and transferred onto a polyvinylidene fluoride (PVDF) membrane and incubated with first antibodies in T-TBS with 1% bovine serum albumin for 12 h at 4 °C. The PVDF membrane was then incubated with secondary antibodies for 1 h at 16 °C. The antibodies used in this study are listed in Table 1. The experimental results were visualized with ELC reagent (Thermo Scientific, Rockford, IL, USA) using the iBright™ Imaging System (Thermo Fisher Scientific, Inc., Waltham, MA, USA). A graph showing relative protein expression normalized using β-actin or inactive form was created.

### Statistical analysis

All data were analyzed using SPSS (version 15.0; IBM Corp., Armonk, NY, USA) and are presented as the mean ± standard deviation (SD). Differences among experimental groups were evaluated using t-test (Figs. 1–3) and one-way analysis of variance (ANOVA), followed by Tukey’s honestly significant difference (HSD) test for post hoc comparisons. Statistical significance levels were set at *p < 0.05, **p < 0.01, and ***p < 0.001. Graphs were generated using Sigma Plot (version 8.0).

## Supplementary information

blot image

## Data Availability

Data will be made available upon request.
