# Correction: Diazinon induces testicular dysfunction and testicular cell damage through increased reactive oxygen species production in mouse

**DOI:** 10.1038/s41420-025-02520-x

**Published:** 2025-05-12

**Authors:** Ran Lee, Won-Young Lee, Dong-Wook Kim, Hyun-Jung Park

**Affiliations:** 1 Department of Livestock, Korea National University of Agriculture and Fisheries, Jeonbuk, Korea; 2 Department of Animal Biotechnology, College of Life Science, Sangji University, Wonju-si, Korea

**Keywords:** Endocrine reproductive disorders, Apoptosis

Correction to: *Cell Death Discovery* 10.1038/s41420-025-02399-8, published online 21 March 2025

The current reference 12. Lei VLC, Leong TI, Leong CT, Liu L, Choi CU, Sereno MI, et al. Phase-encoded fMRI tracks down brainstorms of natural language processing with subsecond precision. Hum. Brain Mapp. 2024;45:e26617) was incorrectly cited by other authors and sources.

It should read: 12. Leong CT, D'Souza UJ, Iqbal M, Mustapha ZA. Lipid peroxidation and decline in antioxidant status as one of the toxicity measures of diazinon in the testis. Redox Rep. 2013;18:155-64. 10.1179/1351000213Y.0000000054.

The original article has been corrected.

