# Supplementary figures and images for: Diazinon induces testicular dysfunction and testicular cell damage through increased reactive oxygen species production in mouse
Source: Cell Death Discov. 2025 Mar 21;11:113. doi: 10.1038/s41420-025-02399-8 (PMC11928526; doi:10.1038/s41420-025-02399-8)

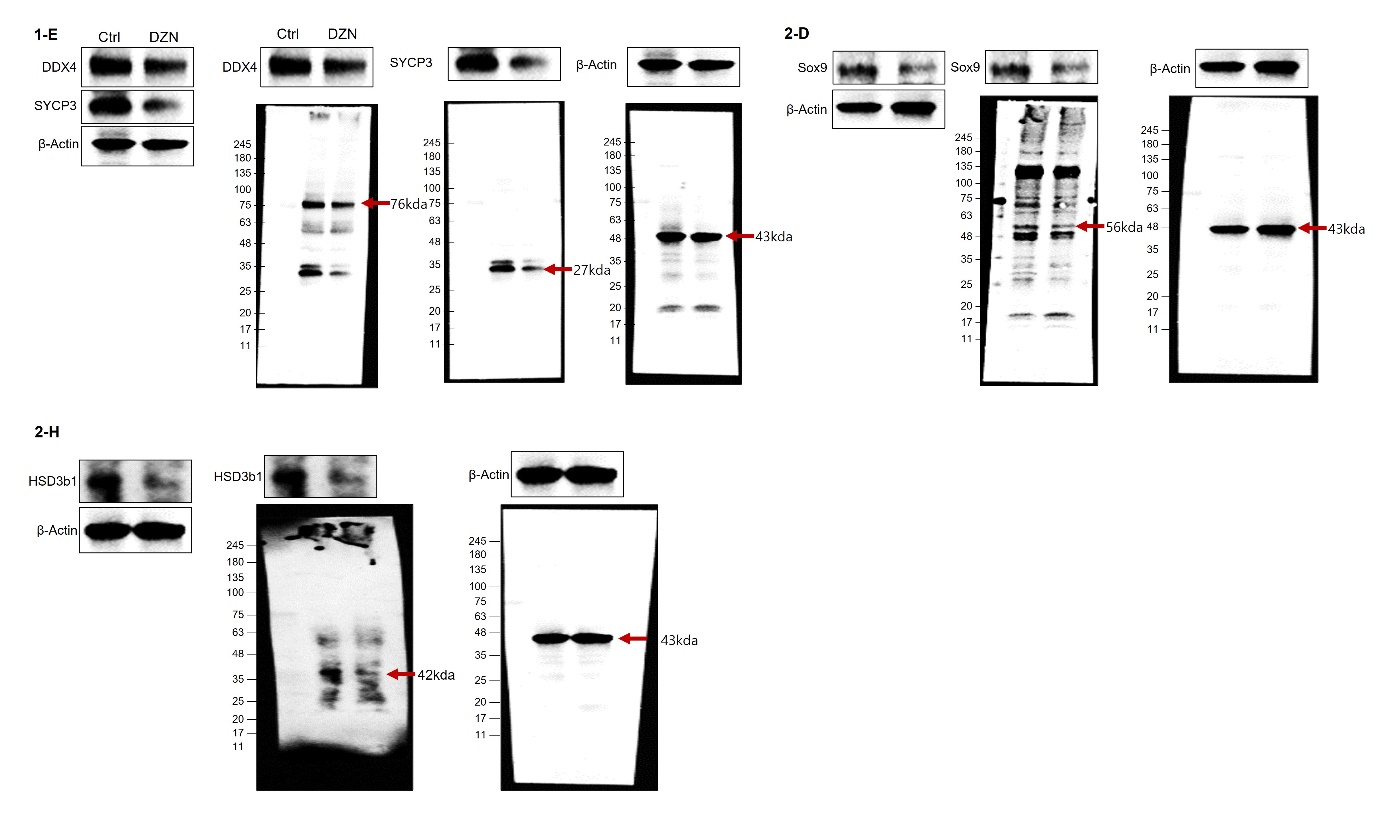

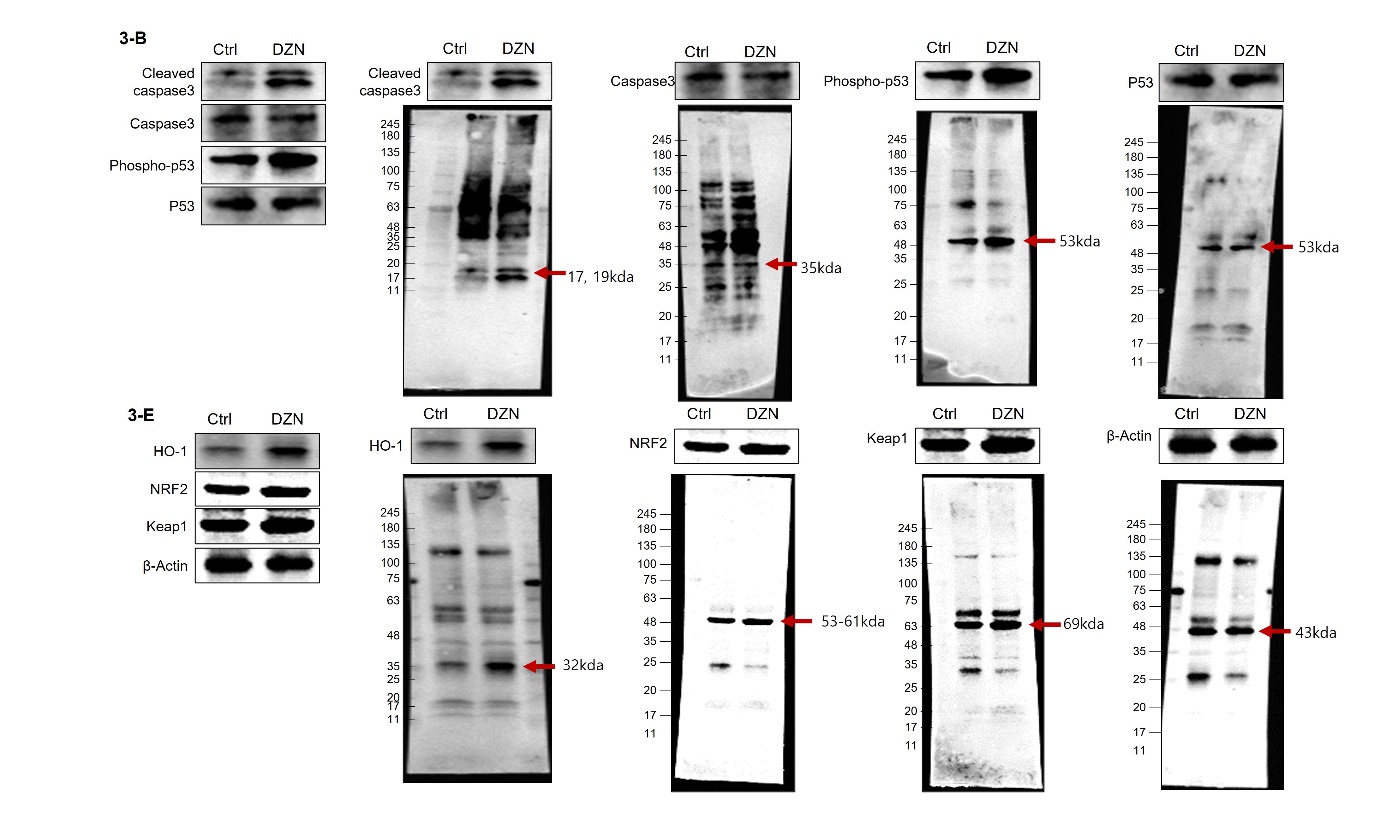
Blot image of each figure


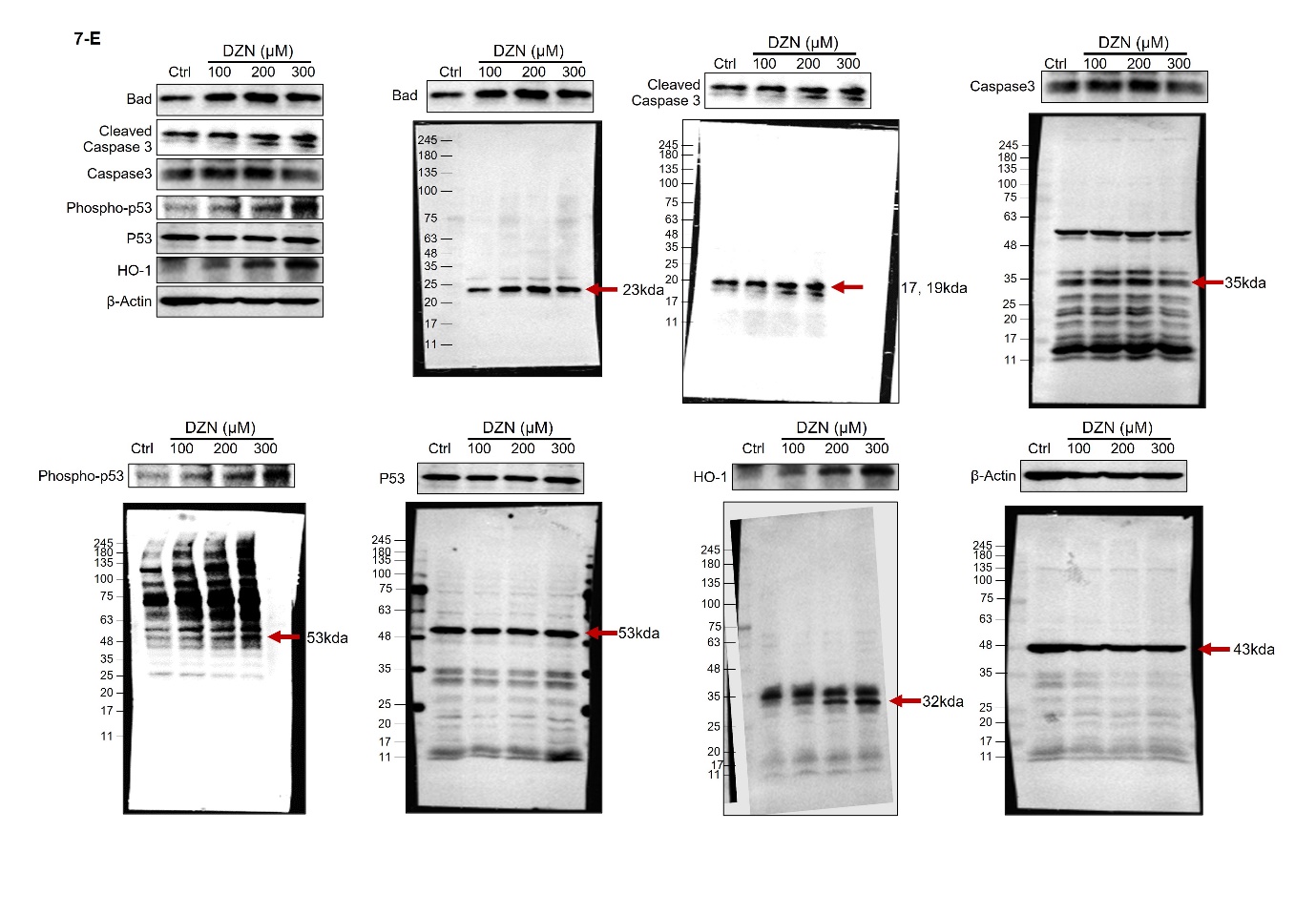

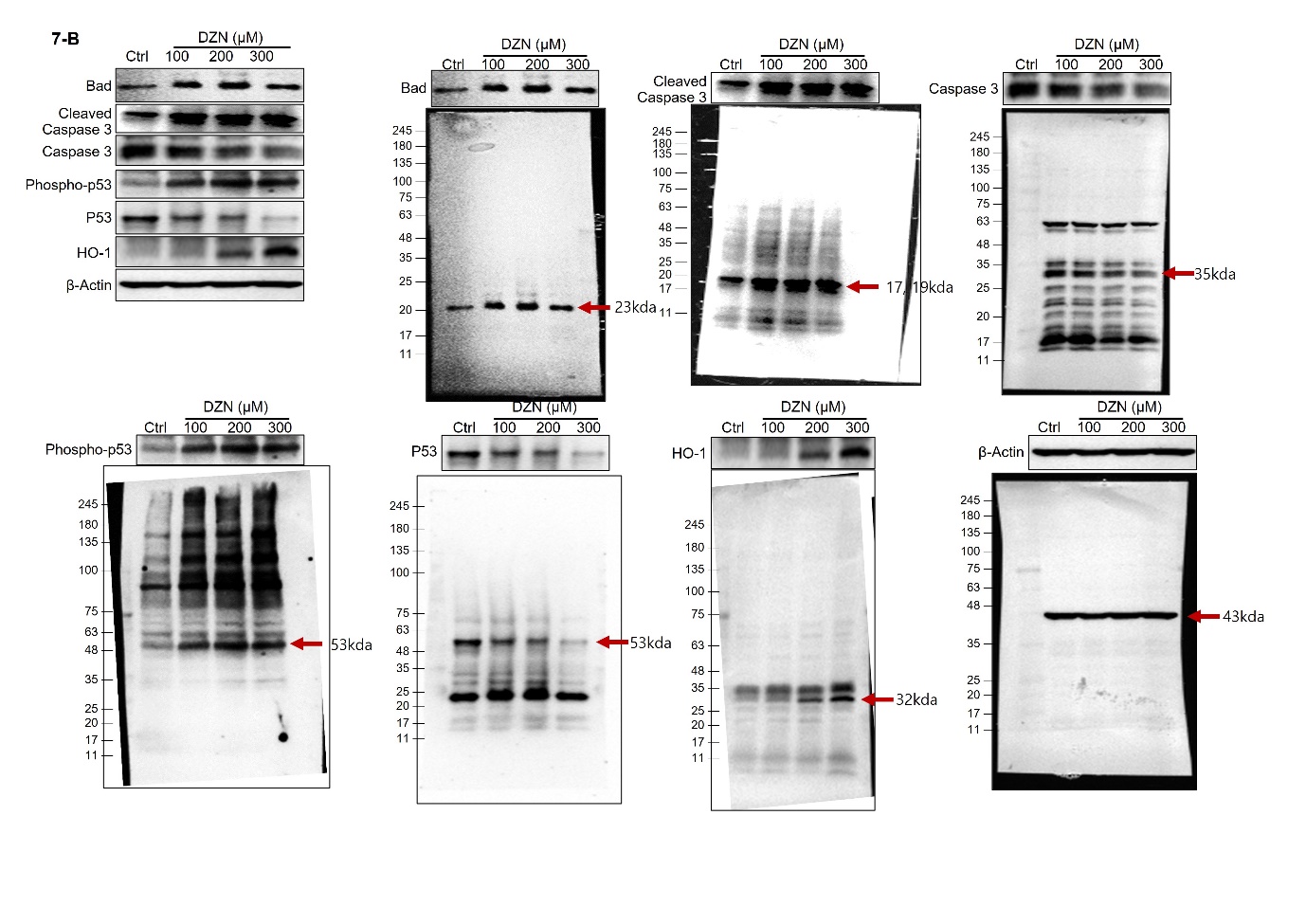


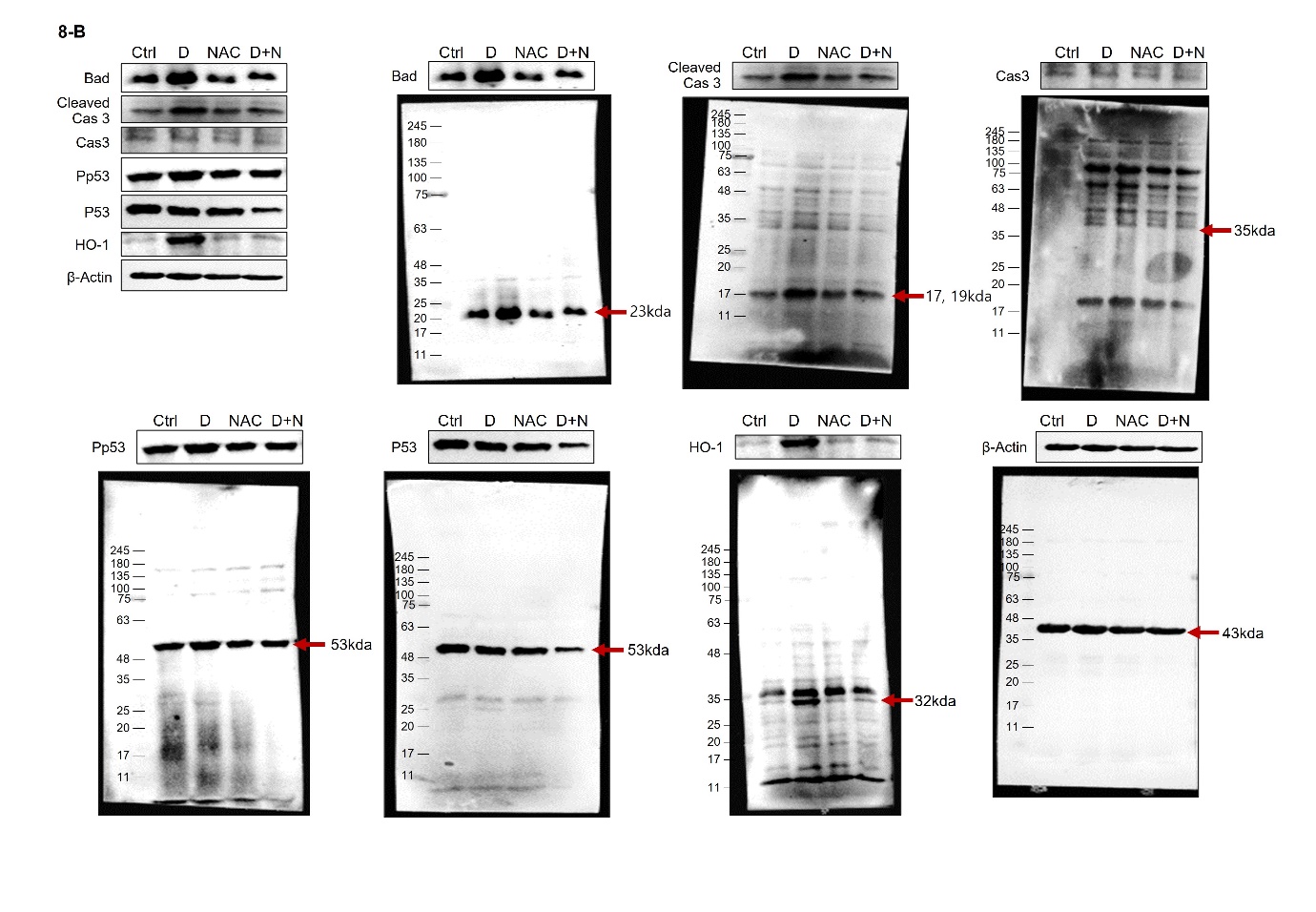

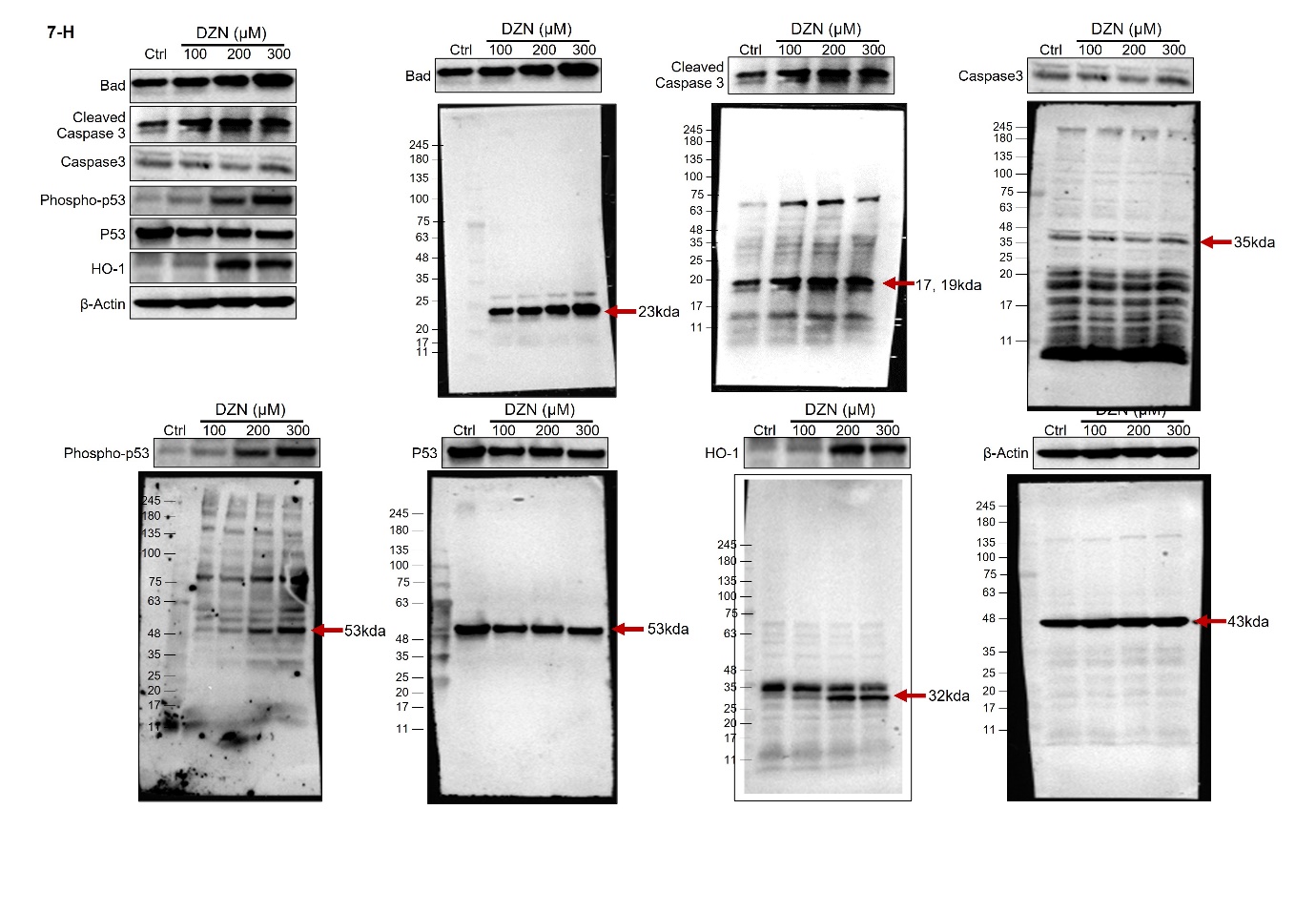


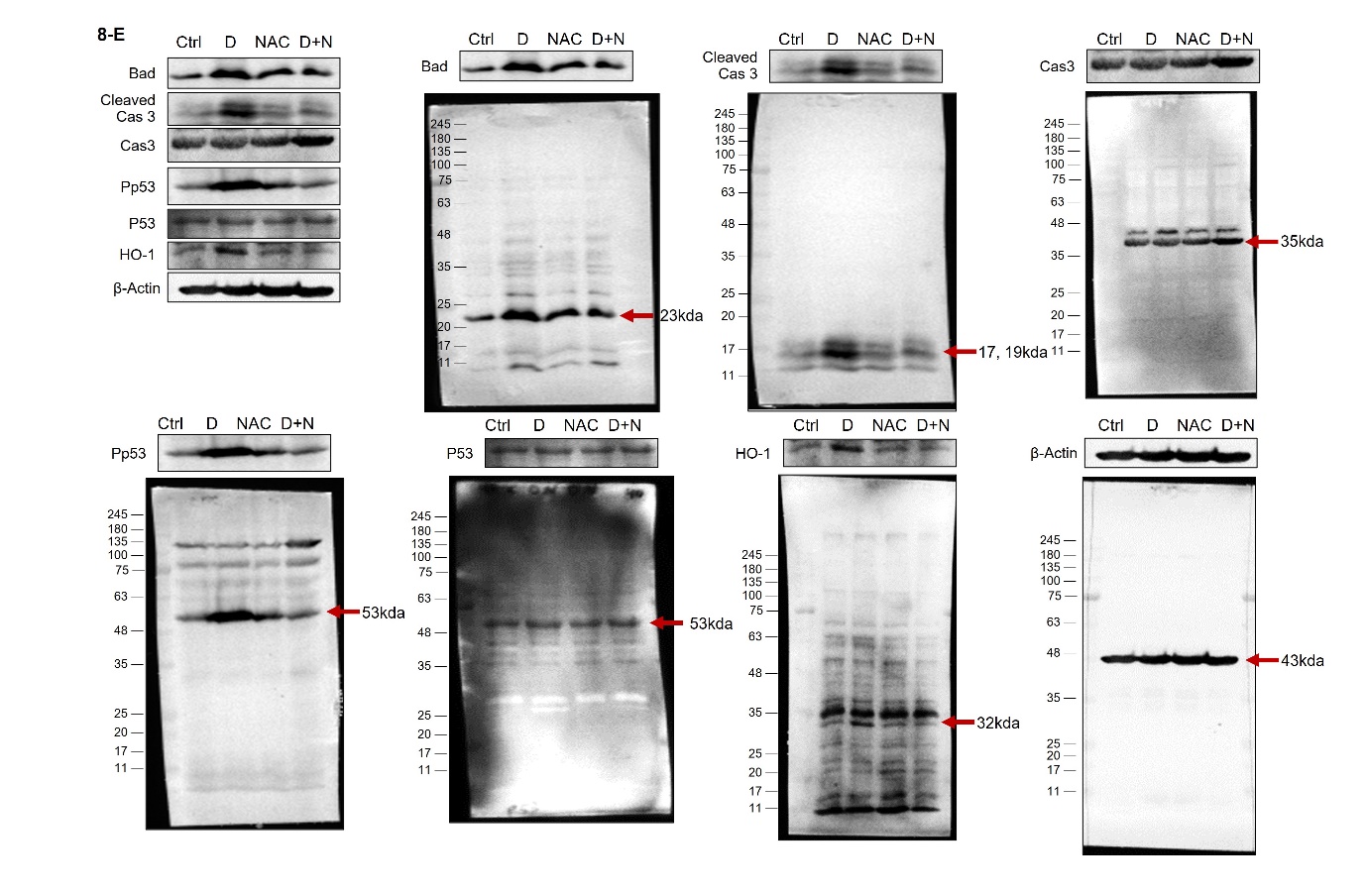

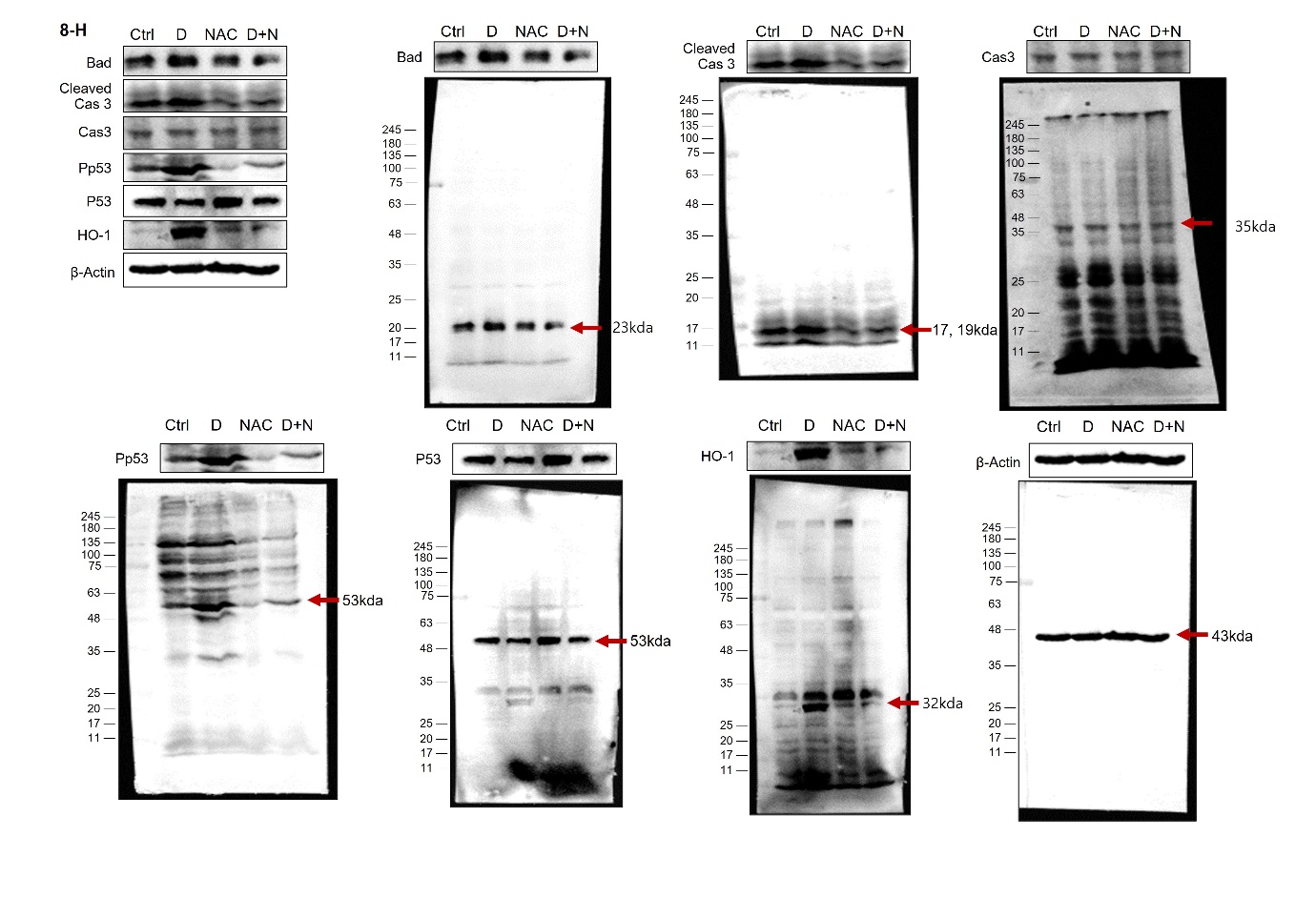

Supplement: Supplementary file 1 — blot image [file 41420_2025_2399_MOESM1_ESM.docx]
